# *PtoMYB031*, the R2R3 MYB transcription factor involved in secondary cell wall biosynthesis in poplar

**DOI:** 10.3389/fpls.2023.1341245

**Published:** 2024-01-17

**Authors:** Feng Tang, Bo Jiao, Meng Zhang, Minghui He, Ruiying Su, Keming Luo, Ting Lan

**Affiliations:** ^1^ Chongqing Key Laboratory of Plant Resource Conservation and Germplasm Innovation, Integrative Science Center of Germplasm Creation in Western China (Chongqing) Science City, School of Life Sciences, Southwest University, Chongqing, China; ^2^ Hebei Key Laboratory of Plant Genetic Engineering, Institute of Biotechnology and Food Science, Hebei Academy of Agriculture and Forestry Sciences, Shijiazhuang, China; ^3^ Key Laboratory of Eco-environments of Three Gorges Reservoir Region, Ministry of Education, School of Life Sciences, Southwest University, Chongqing, China

**Keywords:** secondary cell wall biosynthesis, R2R3-MYB transcription factor, *PtoMYB031*, transcriptional inhibition complex, poplar

## Abstract

**Introduction:**

The biosynthesis of the secondary cell wall (SCW) is orchestrated by an intricate hierarchical transcriptional regulatory network. This network is initiated by first-layer master switches, SCW-NAC transcription factors, which in turn activate the second-layer master switches MYBs. These switches play a crucial role in regulating xylem specification and differentiation during SCW formation. However, the roles of most MYBs in woody plants are yet to be fully understood.

**Methods:**

In this study, we identified and isolated the R2R3-MYB transcription factor, *PtoMYB031*, from *Populus tomentosa*. We explored its expression, mainly in xylem tissues, and its role as a transcriptional repressor in the nucleus. We used overexpression and RNA interference techniques in poplar, along with Yeast two-hybrid (Y2H) and bimolecular fluorescence complementation (BiFC) assays, to analyze the regulatory effects of *PtoMYB031*.

**Results:**

Overexpression of *PtoMYB031* in poplar significantly reduced lignin, cellulose, and hemicellulose content, and inhibited vascular development in stems, resulting in decreased SCW thickness in xylem tissues. Gene expression analysis showed that structural genes involved in SCW biosynthesis were downregulated in *PtoMYB031-OE* lines. Conversely, RNA interference of *PtoMYB031* increased these compounds. Additionally, PtoMYB031 was found to recruit the repressor PtoZAT11, forming a transcriptional inhibition complex.

**Discussion:**

Our findings provide new insights into how PtoMYB031, through its interaction with PtoZAT11, forms a complex that can suppress the expression of key regulatory genes, *PtoWND1A* and *PtoWND2B*, in SCW biosynthesis. This study enhances our understanding of the transcriptional regulation involved in SCW formation in poplar, highlighting the significant role of PtoMYB031.

## Introduction

The biosynthesis of the secondary cell wall (SCW) constitutes an intricate and highly orchestrated process, shaping the unique properties of various plant tissues, most notably wood and fiber. Structurally, SCW consists of multiple layers that are rich in cellulose, hemicellulose, and lignin, conferring rigidity and strength to the plant cells (Zhong et al., 2019). Functionally, SCW plays an indispensable role in various vital functions, including providing mechanical support, facilitating water transport, and offering protection against pathogens. These characteristics not only make SCW pivotal to the overall growth and development of plants but also contribute significantly to the utility of wood and fiber in commercial industries (Sarkanen, 1976; Behr et al., 2019).

The transcriptional network that regulates SCW formation consists of a multilayered cascade of transcription factors (TFs), with the NAC (NAM, ATAF, and CUC) domain proteins functioning as the top-layer regulators (Olsen et al., 2005; Mitsuda et al., 2007; Takata et al., 2019). These Secondary wall *NAC*s (*SWN*s) orchestrate SCW formation by activating second-layer master switches, predominantly within the *MYB* protein family (Zhong et al., 2010b; Nakano et al., 2015; Zhong and Ye, 2015). This *NAC*-*MYB*-based system plays a crucial role in promoting the expression of downstream genes responsible for the biosynthesis of SCW components such as lignin, cellulose, and hemicelluloses (Zhong and Ye, 2014; Taylor-Teeples et al., 2015; Zhang et al., 2018; Chen et al., 2019). In *Arabidopsis*, 10 members of *SWN*s including *VND*s (vascular-related NAC domain domains: *AtVND1-7*) and *NST*s (NAC secondary wall thickening promoting factors: *AtNST1-3*) act as master regulators (Zhong et al., 2010b; Taylor-Teeples et al., 2015).

This *NAC*-*MYB* system has been extensively elucidated in the dicot model organism, *Arabidopsis thaliana*. Studies reveal that in *Arabidopsis*, the *NAC* TFs *SND1*/*NST3* and *NST1* act as master regulators (Zhong et al., 2010b; Taylor-Teeples et al., 2015). *SND1*, with specific expression in interfascicular and xylary fibers, directly binds to the promoter of *MYB46* and *MYB83* through secondary wall *NAC*-binding elements (SNBEs), stimulating their expressions (Zhong et al., 2006; Zhong et al., 2010b). Functioning as pivotal transcriptional switches, *MYB46* and *MYB83* regulate pathways for cellulose, xylan, and lignin biosynthesis. Enhanced expression of these genes facilitates secondary wall formation, while their combined mutations lead to its deficiency, emphasizing their secondary-layer master switch role in SCW biosynthesis (Ko et al., 2012; Zhong and Ye, 2012).

Recent studies have shown that wood formation in poplar is also controlled by transcriptional regulatory networks involving in *NAC* and *MYB* transcription factors. This suggests a conservation of the molecular mechanisms regulating SCW biosynthesis between herbaceous *Arabidopsis* and woody plants (Ye and Zhong, 2015; Chen et al., 2019). A total of 12 *NAC* members of *NST/SND* orthologs, named *WOOD ASSOCIATED NAC DOMAIN TRANSCRIPTION FACTORS* (*WNDs*), have been identified in poplar. All of them are specifically expressed in the xylem, and quadruple mutants of *PtrWND1A/1B/2A/2B* show deficiency in SCW in xylem wood fibers, xylem ray parenchyma cells, and phloem fibers. Overexpression of *PtrWND1B* and *PtrWND2B* can increase the thickness of SCW (Zhong et al., 2010a; Chen et al., 2019; Takata et al., 2019). In wood formation, overexpression of *PtrWND2B* can cause increased expression of *PtrMYB2/3/20/21*, *PtrMYB18/152*, *PtrMYB90/167/161/175*, *PtrMYB26/31/158/189*, which are homologous to *Arabidopsis MYB46/83*, *MYB20/43*, *MYB52/54*, and *MYB69*, respectively (Ye and Zhong, 2015). Both *PtrMYB2* and *PtrMYB21* can promote the biosynthesis of SCW in *Arabidopsis* and poplar, and restore the cell wall defect phenotype of the *myb46 myb83* double mutant (Mccarthy et al., 2010; Zhong et al., 2013).

The *MYB* superfamily, one of the most abundant classes of TFs in plants, plays am indispensable role in SCW biosynthesis (Xiao et al., 2021). Despite the large number of plant-specific *MYB* genes contributing to the evolution of plant-specific physiological or developmental processes, their roles in the SCW biosynthesis regulatory network remain largely undefined. At least 192 R2R3-MYB genes have been predicted in the poplar genome, and the functions of these genes in wood formation are under continuous investigation (Wilkins et al., 2009). Most of the *MYB* transcription factors positively regulate secondary xylem development by promoting lignin synthesis, including *PtoMYB055* (Sun et al., 2020), *PtoMYB074* (Li et al., 2018), *PtoMYB092* (Li et al., 2015), *PtrMYB152* (Wang et al., 2014), *PtoMYB170* (Xu et al., 2017) and *PtoMYB216* (Tian et al., 2013). Only a few MYBs, such as *PdMYB221* and *PtoMYB156*, belonging to the C4 subfamily, have been reported to be transcriptional repressors. Ectopic expression of *PdMYB221* in *Arabidopsis* resulted in reduced SCW thickening in vessel and fiber cells (Tang et al., 2015). Overexpression of *PtoMYB156* dramatically decreased the content of lignin, cellulose, and xylan in transgenic poplar (Yang et al., 2017). Both of these C4 subgroup *MYB* members possess a C-terminally conserved ethylene-responsive element binding factor-associated amphiphilic repression (EAR) motif, which is essential for their repression function. Overexpression of *PtrMYB189* in poplar resulted in a reduction in the SCW thickness of xylem fiber and vessel cells. For *PtrMYB189*, site-directed deletion and mutagenesis of 13 amino acids (277-289, GDDYGNHGMIKKE) at the C terminus of *MYB* indicated the importance of this region in target inhibition (Jiao et al., 2019). Moreover, *PtrMYB161* binds to multiple sets of target genes, allowing it to act as both an activator and a repressor. The balance of the two functions may be important to the establishment of regulatory homeostasis for normal growth and development (Wang et al., 2020). However, the functional roles of numerous poplar *MYB* transcription factors in wood development remain largely unknown.

In addition to the *MYB* transcription factors and their EAR motif-mediated repression in secondary cell wall biosynthesis, the *ZAT* family also plays a significant role in transcriptional regulation through a similar mechanism. *ZAT* family members utilize zinc finger structures to bind DNA and exert repression, often mediated by the EAR motif (Sakamoto et al., 2004; Kagale et al., 2010). The *ZAT* family, belonging to the C2H2 zinc finger protein family, plays crucial roles in plant growth, development, and adaptation to environmental stresses (Wang et al., 2019; Han et al., 2020). Studies have shown that overexpression of *AtZAT10* in *Arabidopsis* not only inhibits growth and development but also enhances resistance to heat, drought, and salt stress (Mittler et al., 2006). Similarly, the overexpression of *ZAT18* has been linked to improved drought resistance (Yin et al., 2017). This similarity in regulatory mechanisms, particularly the reliance on the EAR motif for transcriptional repression, underscores the diversity and complexity of transcriptional control in plant developmental processes.

In this study, we identified a poplar R2R3 MYB transcription factor (TF) gene, *PtoMYB031*, which is preferentially expressed in xylem. Overexpression of *PtoMYB031* in poplar resulted in a reduction in the SCW thickness. *PtoMYB031*, a transcriptional repressor, is localized to the nucleus. We further demonstrated that PtoMYB031 could recruit the repressor PtoZAT11 to form a transcriptional repression complex, thereby inhibiting the biosynthesis of SCW in poplar.

## Materials and methods

### Phylogenetic and molecular evolution analyses

R2R3-MYB protein sequences, derived from *A. thaliana* and *Populus trichocarpa* were extracted from the study Jiang and Rao (2020). These sequences underwent alignment using MUSCLE (Edgar, 2004), followed by refinement with TRIMAL v.1.2 (Capella-Gutiérrez et al., 2009) as documented in **Supplementary Dataset S1** and **S2**. For the phylogenetic analyses depicted in **Supplementary Figures S1** and **1B**, the command line was ‘trimal -in input.phy -out output -htmlout output.html -automated1’. The modelgenerator version 0.84 (Keane et al., 2006) facilitated the estimation of amino acid substitution models, culminating in the selection of JTT+I+G as the optimal model. The phylogenetic trees were constructed using a maximum-likelihood procedure approach implemented in PHYML (Guindon and Gascuel, 2003). One hundred bootstrap replicates were performed in each analysis to obtain the confidence support.

DNASP v.6.12 (Librado and Rozas, 2009) was used to compute the number of nonsynonymous and synonymous substitutions per respective site (*Ka* and *Ks*), and their ratio (*Ka*/*Ks*) for specific duplicated poplar paralogues. These paralogues comprised *PtrMYB026*/*031* and *PtrMYB189*/*158* gene pairs, along with ten proximal protein-coding gene pairs. The temporal context for duplication events was inferred by converting *Ks* values into a time framework, utilizing the equation *T* = *Ks*/2λ with a substitution rate (λ) of 9.1× 10^-9^ specific to *Populus* (Lynch and Conery, 2000; Sterck et al., 2005). Considering the molecular clock of *Populus* operates at approximately one-sixth the pace of *Arabidopsis*, a recalibration was paramount (Tuskan et al., 2006). We adjust the estimated gene duplication rate for *Populus* by applying a correction factor of 65/13 (that is 65 Mya to 13 Mya), reflecting its slower evolutionary rate relative to *Arabidopsis*.

### Conservation and collinearity analysis

Previous analysis uncovered paralogous segments resulting from the salicoid duplication event (Tuskan et al., 2006). In this study, we mapped gene pairs *PtrMYB026*/*031* and *PtrMYB189*/*158* within the *P. trichocarpa* genome. To investigate the mechanisms for the formation of the gene pairs, several specific criteria were considered: (1) Each pair of duplicated genes was located within paralogous segments, originating from the salicoid duplication. (2) The genomic regions containing the target genes demonstrated a high degree of synteny. (3) Phylogenetic analysis indicated that these paralogs represented the most recently divergence. (4) The estimated divergence time was consistent with the salicoid duplication event, occurring approximately 60-65 million years ago.

Conservation analysis of specific DNA sequence segments was conducted using PipMaker (Schwartz et al., 2000). This analysis encompassed the aforementioned gene pairs and included an examination of regions spanning 60-kb both upstream and downstream of these sequences. WebLogo was employed for graphical depiction, representing the conservation across protein sequences (Crooks et al., 2004).

### Gene cloning and vector construction

The complete coding sequence of *PtoMYB031* was amplified using cDNA of *P.tomentosa* by gene-specific primer pairs (**Supplementary Table S1**). The polymerase chain reaction (PCR) protocol initiated with a pre-denaturation phase at 94°C for 5 min. Then by 35 cycles, each consisting of 30 s at 94°C, 30 s at 60°C, and 60 s at 72°C, and followed by a final elongation stage at 72°C for 10 min. Following amplification, PCR products were integrated into the plant binary vector pCXSN (Chen et al., 2009) utilizing an authentic TA cloning kit, and subsequent sequencing was conducted. Furthermore, promoter fragments spanning 2-kb for *Pro-PtoMYB031* and *Pro-PtoWND1A/2B* were amplified from *P. tomentosa* genomic DNA. These fragments were subsequently incorporated into the pCXGUS-P vector, each driving the GUS reporter gene, as delineated in Chen et al. (2009).

### Generation of transgenic plants and growth conditions

*P. tomentosa* underwent stable transformation *via Agrobacterium*-mediated leaf disks infiltration, adhering to methodologies established in prior studies (Jia et al., 2010). Identification of positively transformed poplar lines was achieved through PCR analysis, with specific primers targeting hygromycin- or kanamycin-resistance genes (**Supplementary Table S1**). Poplar plants were cultivated in greenhouse conditions, maintaining a temperature of 25°C. Environmental controls included a photoperiod regimen of 16/8 h (day/night), bolstered with supplemental illumination (4500 lux), and sustained relative humidity at 55%.

### Transcriptional analysis and Quantitative real-time PCR

Expression profiles of R2R3-MYB genes, across diversified tissues and developmental stages, were analyzed using RNA-seq datasets, sourced from the JGI Plant Gene Atlas *via* Phytozome (https://phytozome-next.jgi.doe.gov/geneatlas/) (Sreedasyam et al., 2023) and the PlantGenIE platform (https://plantgenie.org/exPlot) (Sundell et al., 2015). A focused analysis concerning Clade V *MYB* genes within the context of wood biosynthesis was conducted utilizing the high-spatial-resolution RNA-Seq data from AspWood (Sundell et al., 2017). Discrepancies in gene expression across various tissues were elucidated by contrasting normalized FPKM (Fragments Per Kilobase of Transcript Per Million Mapped Reads) values. Data consolidation ensued, culminating in the visual representation via a heat map, facilitated by TBtools v2.012 (Chen et al., 2023).

First-strand cDNA synthesis was synthesized employing the PrimeScript RT reagent Kit (Takara, Dalian, China) according to the manufacture’s instruction. qRT-PCR was conducted utilizing an Mx3000P™ Real-Time PCR System (Agilent Stratagene, USA), using the SYBR-Green PrimeScript RT-PCR Kit (Takara, Dalian, China). Reactions, conducted in 20μL volumes, comprised 10 μL SYBR, 0.4 μL ROX, 6.8 μL ddH_2_O, 2 μL diluted template, and 0.4 μL of each primer, targeting specific genes. Three technical replicates were taken for each biological sample. The *PtoUBQ* gene was used as an internal control, transcript levels were normalized against the average expression of the *PtoUBQ* gene. Relative expression data were calculated using the 2-ΔΔCт method. Comprehensive details pertaining to the primers employed in qRT-PCR are accessible in **Supplementary Table S1**.

### GUS staining

The positive transgenic lines containing the *GUS* reporter gene driven by the *PtoMYB031* promoter were identified. Cross-section of stems and leaves underwent fixation in acetone for one hour at 20°C, succeeded by two wash cycles in double-distilled H_2_O (ddH_2_O). Subsequently, the samples were incubated in staining buffer in darkness at 37°C for 3 h. Chlorophyll extraction was performed using a destaining solution (ethanol: acetic acid, 3:1 ratio) at room temperature for 30 min, followed by two ddH_2_O rinses. The chlorophyll-free stained materials were then scrutinized under an Olympus SZX16 microscope, and digital imaging was conducted.

### Transcriptional activation analysis and subcellular localization

The coding sequence of *PtoMYB031* was incorporated into the pGBKT7 vector (Clontech, Shiga, Japan), followed by the transformation of this recombinant plasmid into *Saccharomyces cerevisiae* Gold2 by the LiAc/PEG method (Gietz and Schiestl, 2007). Transformed yeast cells were cultured on SD/-1 medium lacking tryptophan (Trp) to select positive clones, subsequently relocating them to SD/-3 medium, deficient in Trp, histidine (His) and adenine (Ade) for the transactivation assay. Additionally, X-α-gal was used for filter-lift assays, elucidating the transcription activation activity of *PtoMYB031*. The coding sequence of *PtoMYB031* was aligned with the CaMV 35S promoter and the Tnos terminator within the pCX-DG plant binary vector (Chen et al., 2009). The GFP-MYB031 fusion protein was introduced into *Agrobacterium tumefaciens* strain EHA105 through the freeze–thaw method. Subsequent infiltration into tobacco (*Nicotiana benthamiana*) leaf epidermal cells with the 35S:GFP-MYB031 construct followed methodologies established by Sparkes et al. (2006). After 24-48 h of infiltration, the leaf epidermal cells were examined for green fluorescence signal with a confocal laser microscope (Olympus FV1000, Tokyo, Japan), and also stained with 4’, 6-diamidino-2-phenylindole (DAPI).

### Transient transactivation assays

The transcriptional activity of *PtoMYB031* was examined by amplifying the promoters of *PtoWND1A* and *PtoWND2B* using gene-specific primers. These promoter fragments were cloned upstream of the *GUS* gene in the pCXGUS-P vector (Chen et al., 2009). Subsequently, *A. tumefaciens* EHA105 was transformed with these constructs. Co-transformation of the effector and reporter constructs was then performed on *N. benthamiana* leaves. Post-transformation, the plants were incubated in darkness for 12 h, and then exposed to light cultivation for 2 days, GUS activity was subsequently quantified using fluorescence spectrophotometry (Hitachi F-7000, Tokyo, Japan). Protein concentrations were measured by the Bradford method (Bradford, 1976).

### Scanning electron micrograph assays

For SEM analysis, the 8^th^ internode stems from three-month-old poplar plants were harvested. Prepared specimens were placed in an SEM chamber and examined under Backscatter Electron (BSE) mode at an acceleration voltage of 15 kV. Observations were conducted using a Phenom Pro microscope (Eindhoven, The Netherlands) in adherence to the manufacturer’s instructions. Digital captures of the images were executed, ensuring reproducibility with three biological replicates.

## Results

### *MYB026/031* as a potential key regulator in poplar wood development

Comprehensive phylogenomic analyses of plant R2R3-MYB TFs have previously delineated the presence of 10 distinct subfamilies of R2R3-MYB proteins across land plants (Jiang and Rao, 2020). We identified key *MYB* genes influencing poplar stem development by analyzing 190 sequences from Jiang and Rao (2020), conducting phylogenetic reconstruction, and correlating with tissue expression profiles from the *Populus* Gene Atlas (Phytozome v13.0, Sreedasyam et al., 2023). Our phylogenetic analysis revealed that the poplar *R2R3-MYB* genes clustered into 10 clades, with Clade VIII is the largest (**Supplementary Figure S1**), corroborating earlier discoveries (Jiang and Rao, 2020). A total of 14 *MYB* genes (10 and 4 in Clade VIII and Clade V, respectively) showed specifically expressed in stem (labelled by a red asterisk in **Supplementary Figure S1**). This specificity in expression intimates a critical involvement in the stem’s architectural and functional development within poplar.

**Figure 1:**
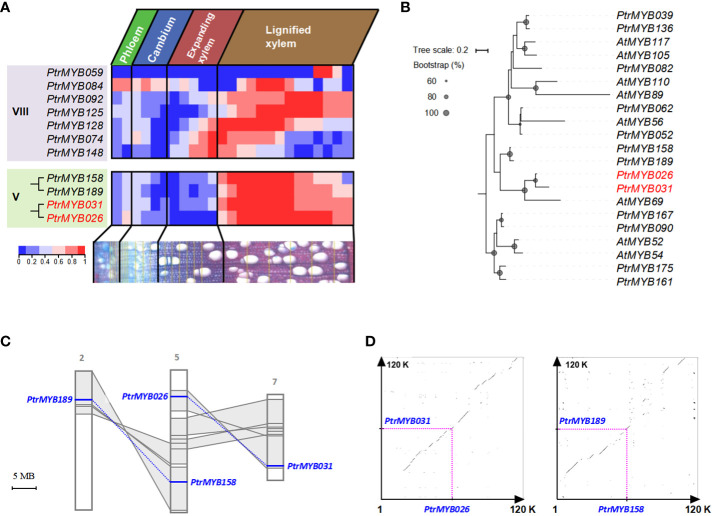
The evolutionary mechanisms of *PtrMYB026*/*MYB031* gene pair in poplar and their expression patterns. **(A)** Expression heatmap of selected *MYB* genes the developing secondary vascular tissues of poplar, base on data from the AspWood database. This heatmap depicts normalized expression levels, enabling a clear comparison of the same *MYB* gene across different stages of wood formation in poplar. **(B)** Phylogenetic analysis of *MYB* genes from Clade VIII and Clade V, exhibiting specific expression in the stem. All the sequences are documented in **Supplementary Dataset S2**. **(C)** Distribution of *PtrMYB189/158* and *PtrMYB026/031* gene pairs. Homologous genome segments are marked in grey, with connecting lines denoting gene pairs. **(D)** Syntenic analysis of genomic regions flanking these gene pairs.

We used the AspWood database to analyze transcript abundance in aspen’s secondary xylem and phloem, providing exact timing for gene expression changes (Sundell et al., 2017). Within this database, 11 out of the 14 identified *MYB*s were recognizable: the quartet from Clade V manifested a conspicuous expression zenith in lignified xylem tissues, whereas their Clade VIII counterparts exhibited a more diversified expression terrain (**Figure 1A** and **Supplementary Figure S2**). Notably, specific Clade VIII members have garnered attention for their roles in lignin biosynthesis during secondary cell wall formation in poplar, as documented in recent studies (Balmant et al., 2020; Liu et al., 2021). Within Clade V, *PtrMYB189/158* and *PtrMYB026/031* were divided into two distinct groups with high bootstrap support (**Figure 1B**; **Supplementary Figure S1**, the gene names *PtrMYB189/158* and *PtrMYB026/031* refer to genes identified in the *Populus trichocarpa* genome). Whereas *PtrMYB189*’s predominance in secondary vascular tissues and its repressive influence on secondary cell wall biogenesis have been illuminated (Jiao et al., 2019). *PtrMYB026/031*, despite its implicated significance in poplar’s secondary wall, continues to puzzle researchers, its exact regulatory mechanisms remaining elusive. This investigation, therefore, pivots on decoding the intricate regulatory paradigms tethered to *PtrMYB026/031* within clade V.

### Formation of the *MYB026/MYB031* gene pair by the salicoid duplication event

We investigated the distribution of the *PtrMYB026*/*031* and *PtrMYB189*/*158* gene pairs within the *Populus* chromosomes. Chromosome 5 accommodates *PtrMYB026* and *PtrMYB158*, whereas *PtrMYB189* and *PtrMYB031* were located on chromosomes 2 and 7, respectively. The paralogous chromosomal segments created by the recent whole-genome salicoid duplication event (60-65 million year ago) (Tuskan et al., 2006) are highlighted in gray and connected with lines (**Figure 1C**). Notably, *PtrMYB026*/*031* and *PtrMYB189*/*158* are each located in a pair of paralogous blocks, suggesting their likely emergence from this salicoid duplication. An extended synteny analysis encompassing 60-kb regions flanking *PtrMYB026*/*031* and *PtrMYB189*/*158* affirmed a pronounced synteny within these genomic locales (**Figure 1D**).

We calculated the divergence timelines for the *PtrMYB026*/*031* gene pair and ten adjacent paralogs. The deduced timeframe for these duplication events spans from roughly 43.35 to 99.18 Mya, with an average of 65.38 Ma. Concurrently, for *PtrMYB189*/*158* and their neighboring paralogs, the average value is approximately 65.25 Mya (**Supplementary Table S2**), congruent with the era of salicoid duplication. Collectively, phylogenetic and syntenic results indicated that *PtrMYB026*/*031* and *PtrMYB189*/*158* were born from the whole-genome duplication event in the Salicaceae, ~ 60 to 65 Mya.

Previous studies showed that gene pairs, born through whole-genome duplications, often preserve analogous functions (Lan et al., 2009). This preservation is potentially attributable to the large-scale segmental duplication’s replication of numerous genes, inclusive of their regulatory sequences (Casneuf et al., 2006; Kim et al., 2006). Scrutinizing the expression dynamics of *PtrMYB026* and *PtrMYB031*, we discerned a steadfastly superior expression profile for *PtrMYB031* across diverse tissues relative to *PtrMYB026* (**Supplementary Figure S3**). This differential suggests *PtrMYB031*’s heightened significance in orchestrating developmental sequences. Thus, our ensuing investigative efforts will concentrate on an exhaustive dissection and elucidation of *PtrMYB031*’s regulatory capacities.

### Analysis of *PtoMYB031* gene expression, protein localization, and transcriptional activation

Due to the establishment of a mature genetic transformation system in *Populus tomentosa*, we identified and cloned the ortholog of *MYB031* in the *Populus tomentosa* genome for further analysis. A significant homology was observed among the Clade V *MYB* genes. The analysis revealed a characteristic R2R3 domain at the N-terminal of *PtoMYB031*, which comprises 348 amino acids. This includes the R2 region (22-62 aa) and the R3 region (85-125 aa) (**Supplementary Figure S4**). Accordingly, *PtoMYB031* can be categorized as a prototypical R2R3-type MYB transcription factor.

**Figure 2:**
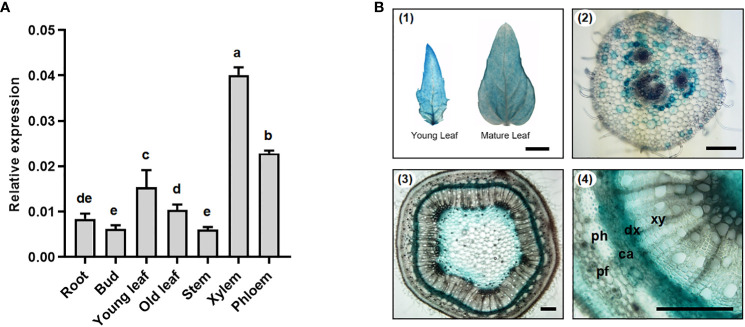
Expression patterns of *PtoMYB031* in *P. tomentosa*. **(A)** The relative expression level of *PtoMYB031* in various tissuess *via* RT-PCR. Error bars represent standard deviation (± SD) from three biological replicates. **(B)** GUS activity histochemical analysis in pMYB031::GUS transgenic poplar plants, including young and mature leaves (1), petiole cross-sections (2), and stem cross-sections (3, 4). Scale bars correspond to 1cm (1), 100 μm (2, 3) and 200 μm (4). Different lowercase letters indicate significant differences as tested by One-way ANOVA followed by Tukey’s test.

To ascertain the tissue-specific expression of *PtoMYB031*, particularly in the stems, a quantitative real-time PCR (qRT-PCR) was conducted. This study examined the expression levels of *PtoMYB031* across various tissues of *Populus tomentosa*, encompassing roots, bud, leaves (both juvenile and mature), and the stem’s 7^th^ internode, specifically focusing on its xylem and phloem. Notably, enhanced expression was detected in the xylem (**Figure 2A**). To further investigate its tissue expression specificity, the *PtoMYB031* promoter was cloned into a vector hosting the *GUS* reporter gene. GUS staining demonstrated *PtoMYB031* expression in the xylem-differentiating area of the cambium, the parenchyma cells neighboring the phloem in the petiole, and within the leaves (**Figure 2B**). These indicates a potential role of *PtoMYB031* in xylem differentiation and maturation.

Earlier research highlighted that *PtrMYB161* and *PtrMYB189* (Clade V members) participate in wood development, acting as transcriptional repressors (Jiao et al., 2019; Wang et al., 2020). To evaluate the transcriptional activation potential of *PtoMYB031*, its coding sequence was cloned into the pGBKT7 yeast vector. Growth on SD/-3 medium containing α-galactoside resulted in the development of a blue colony (**Supplementary Figure S5A**), suggesting that *PtoMYB031* may have the capability to initiate reporter gene expression in this yeast system. For determining the subcellular localization of the *PtoMYB031* protein, its coding sequence was fused with the *GFP* reporter gene and transiently transformed into tobacco leaf epidermal cells. Observations made post 24-48 h of culture using confocal laser microscopy exhibited fluorescence predominantly in the nucleus (**Supplementary Figure S5B**). This evidence underlines that *PtoMYB031* is a nuclear protein. In conclusion, our findings support the notion that *PtoMYB031* is a nuclear-localized R2R3-type activator.

### Impact of *PtoMYB031* manipulation on poplar growth and morphology

**Figure 3:**
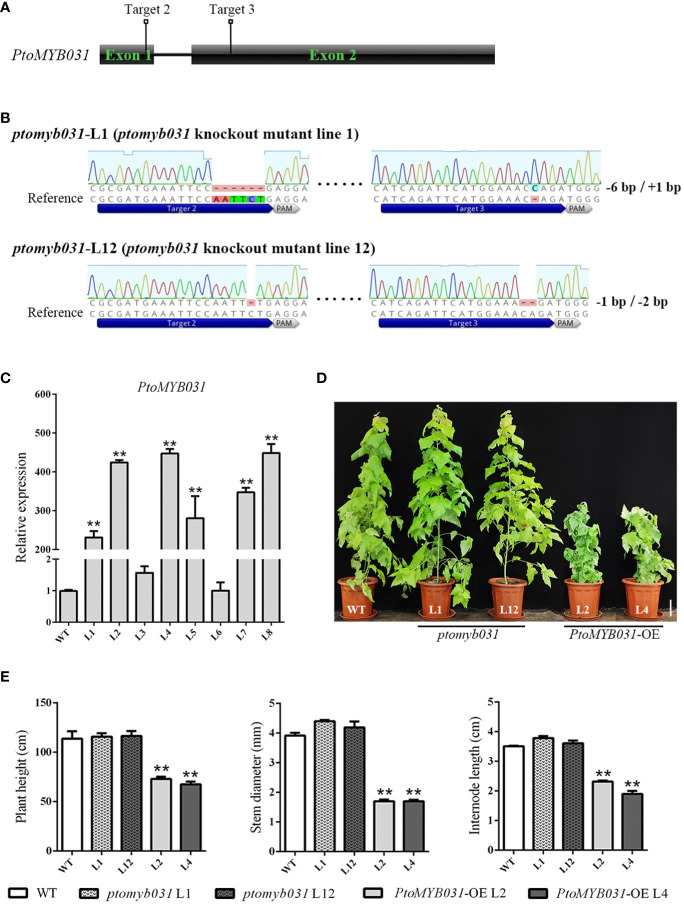
Generation and analysis of *PtoMYB031* overexpressing (OE) and *ptomyb031* mutant lines. **(A)** Schematic of gene architecture highlighting sgRNA target sites. **(B)** Mutations at the target site for *PtoMYB031* mutants, which were generated using the CRISPR-Cas9 system with nucleotide deletion or insertion. The dashed lines represent deletions, with the reference sequence shown. **(C)** The expression level of *PtoMYB031* in wide-type (WT) and *PtoMYB031-OE* lines (L1-L8). The values of WT were normalized to 1. **(D)** Morphological comparison of 3-month-old WT, *ptomyb031* mutants, and *PtoMYB031-OE* lines. Scale bar: 10 cm. **(E)** Statistical analysis of height, stem diameter, and internode length in *PtoMYB031-OE* lines versus WT, with error bars showing standard deviation (± SD) from three replicates. Statistical significance determined by Student’s t-test (** *p* < 0.01).

To investigate the function of *PtoMYB031* in the wood development process of poplar trees, we constructed both knock-out and overexpression vectors for *PtoMYB031*. We transformed *P. tomentosa* with the constructed *PtoMYB031* overexpression (*PtoMYB031*-OE) and *PtoMYB031* knock-out vectors, and subsequently identified the transgenic lines. The results indicated that genome editing occurred at both the second and third target sites, yielding two knock-out lines, *ptomyb031*-L1 and *ptomyb031*-L12. Specifically, *ptomyb031*-L1 exhibited a 6 bp deletion at the second target site and a 1 bp insertion at the third target site, while *ptomyb031*-L12 had a 1 bp deletion at the second target site and a 2 bp deletion at the third target site (**Figures 3A, B**), lead to a change in the amino acid sequence that results in the production of truncated proteins (**Supplementary Figure S6**). For the overexpression lines, six were identified, and two lines (L2 and L4) were selected for subsequent experimental analyses. We observed that the expression level of *PtoMYB031* in L2 and L4 was elevated approximately 400-fold compared to the wild-type (WT) (**Figure 3C**).

Compared to the WT, the growth of *PtoMYB031*-OE plants was significantly inhibited, displaying characteristics such as dwarfism (**Figure 3D**), uneven leaf surfaces, and upward curling of leaf edges (**Supplementary Figure S7**). The results further demonstrated that the plant height in the *PtoMYB031*-OE lines was reduced by approximately 36% (**Figure 3E**), stem diameter by approximately 55%, and internode length by approximately 41%, compared with the WT (**Figure 3E**). In contrast, the knock-out lines did not exhibit noticeable changes (**Figures 3D, E**). We hypothesize that the absence of a conspicuous phenotype in the knock-out lines may be attributed to functional redundancy between *PtrMYB026* and *PtrMYB031*. It is plausible that *PtrMYB026* could compensate for the loss of function in *PtrMYB031*, thereby mitigating any significant alterations in the overall phenotype.

To elucidate the phenomenon of leaf irregularity in transgenic plants, we conducted phloroglucinol staining of the leaves and veins. Our findings revealed that the *PtoMYB031*-OE plants exhibited a significantly reduced number of veins compared to the wild-type, and the vascular bundle development in the petiole was markedly delayed (**Supplementary Figure S8A**). Subsequently, we employed toluidine blue staining to assess the primary and secondary veins of the leaves. The analysis indicated that vascular development in the veins of *PtoMYB031*-OE lines was notably slower than in the WT. Additionally, the angle formed between the leaf and the midvein in the *PtoMYB031*-OE lines was smaller compared to that in the WT (**Supplementary Figure S8B**). Collectively, these findings suggest that the vascular bundle development in the leaves of *PtoMYB031*-OE plants is impeded, implying a potential inhibitory role of *PtoMYB031* in leaf vascular development.

### Inhibition of xylem and secondary cell wall development in poplar stem by overexpression of *PtoMYB031*

**Figure 4:**
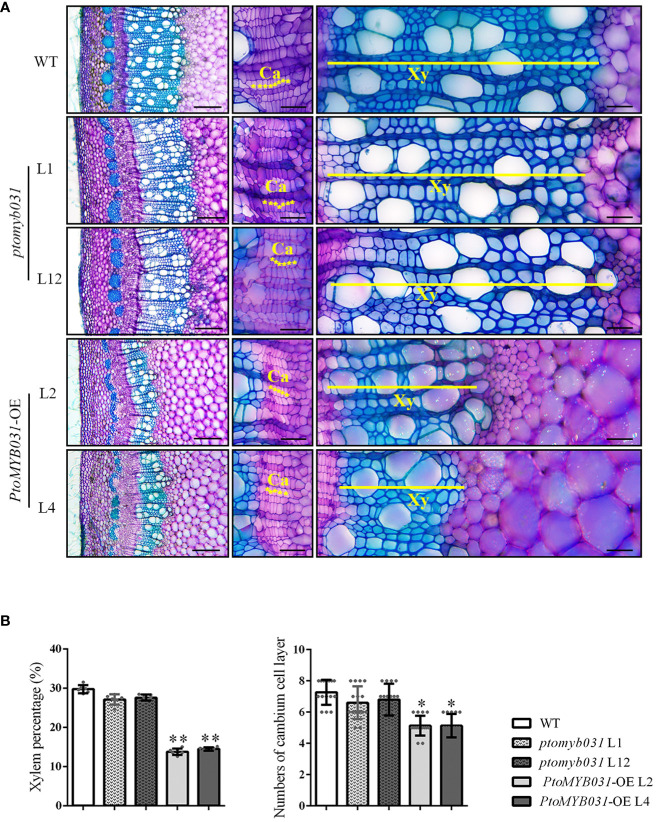
Microscopic analyses of stem in *ptomyb031*, *PtoMYB031-*OE lines, and WT. **(A)** Cross sections of stem in WT, *ptomyb031* (L1 and L12), and *PtoMYB031-*OE lines (L2 and L4) were taken from the eighth internode of 3-month-old plants. The yellow asterisks represent the cambium (Ca), yellow straight line represent the xylem (Xy). Scale bar: 200 μm (left column), 40 μm (right two columns). **(B)** Quantitative assessment of xylem area and cambium cell layers, with statistical significance evaluated using Student’s t-test (* *p* < 0.05, ** *p* < 0.01).

We examined the 8^th^ internode stems (counted from the apex) of *PtoMYB031*-OE lines (L2 and L4) and *ptomyb031* mutants (L1 and L12), subjecting them to sectioning and toluidine blue staining. In the *PtoMYB031*-OE lines, the proportion of xylem was significantly reduced, and the number of cambial cells revealed a slight decrease in the OE lines (**Figures 4A, B**). Significant changes were also noted in the development of pulp and phloem tissues (**Supplementary Figure S9A**). In the *PtoMYB031*-OE lines, there was a marked reduction in the area of both pulp and phloem (**Supplementary Figure S9B**). However, relative to the dramatic alterations observed in the xylem, these changes did not result in a significant alteration in the proportion of pulp and phloem within the overall stem cross-section (**Supplementary Figure S9B**). This indicates that overexpression of *PtoMYB031* significantly alters the quantity of different cell types within the stem, especially the xylem, thereby substantially impacting the overall structure of the stem. Furthermore, in the *PtoMYB031*-OE lines, the lignin autofluorescence signal was weaker than that of WT (**Supplementary Figure S10**), and have significantly thinner cell walls (**Supplementary Figure S11**). These findings indicate that the overexpression of *PtoMYB031* may negatively regulate the secondary growth of poplar stem, and inhibit the synthesis of secondary cell walls in the xylem of *P. tomentosa*. Compared to the wild type, no significant alterations were observed in the xylem development of the *ptomyb031* mutant. The lack of apparent phenotypic differences in the knockout lines may be attributed to functional redundancy between *PtoMYB031* and *PtoMYB026*.

To further validate *PtoMYB031*’s functions, we generated a *PtoMYB031*-RNAi vector and transformed it into *P. tomentosa*. The down-regulation of *PtoMYB031* led to a slight and significant increase in stem thickness for the L2 and L8 lines, respectively (**Supplementary Figure S12A**). However, it did not have a substantial impact on either plant height or the proportion of xylem area (**Supplementary Figures S12A–C**). Notably, sections of the 8^th^ internode revealed ectopic lignin deposition in the pith of the *PtoMYB031*-RNAi line (**Supplementary Figure S12D**), implying that reduced expression of *PtoMYB031* can promote lignin synthesis. In observations of the overall stem morphology in *PtoMYB031* knockout lines, we encountered similar findings. However, electron microscopic analysis of xylem cell walls in these knockout lines revealed a significant increase in cell wall thickness compared to the WT (**Supplementary Figure S11**). This observation further corroborates the role of *PtoMYB031* in modulating cell wall composition and structure. Mirroring the situation observed in the knockout or RNAi plants, we hypothesize that the absence of a pronounced phenotype in the *PtoMYB031*-RNAi lines may be attributed to the compensatory redundant function of *PtrMYB026*, which could offset the loss incurred by the down-regulation of *PtoMYB031*, thereby mitigating the expected morphological changes.

Subsequently, the contents of secondary cell wall components, including lignin, cellulose, and hemicellulose, were assessed. We found substantially lower levels in *PtoMYB031*-OE lines (L2 and L4) compared to the WT (**Table 1**). In contrast, the *PtoMYB031*-RNAi line (L8) exhibited a slight increase in the total content of lignin, cellulose, and hemicellulose compared to the WT. Thus, reduced expression of *PtoMYB031* appeared to enhance the synthesis of these components in the secondary cell wall (**Table 2**). While these alterations were not as pronounced as in the overexpression lines, we did observe modifications in cell wall morphology and composition in the *PtoMYB031* knockout or RNAi lines.

**Table 1:** Lignin, cellulose, hemicellulose and pectin contents in *PtoMYB031*-OE lines.

	WT	35S:PtoMYB031	
		L2	L4
Lignin			
Acid-soluble	3.31 ± 0.06	3.21 ± 0.13	3.63 ± 0.12
Acid-insoluble	16.53 ± 0.31	13.47 ± 0.29* **-18.51%**	12.40 ± 0.41* **-24.98%**
Total lignin	19.83 ± 0.28	16.69 ± 0.26* **-15.83%**	16.03 ± 0.46* **-19.16%**
Polysaccharide			
Cellulose	47.37 ± 3.11	31.33 ± 1.26** **- 33.85%**	25.64 ± 1.50** **- 45.87%**
Hemicellulose	12.23 ± 1.12	8.03 ± 0.37** **- 34.36%**	5.35 ± 2.53** **- 56.29%**
Pectin	2.98 ± 0.29	4.11 ± 0.20** **+ 37.91%**	4.31 ± 0.40** **+ 44.88%**

Significance is tested via Student’s t-test, where ‘*’ indicates *p* < 0.05 and ‘**’ indicates *p* < 0.01.

The bold values represent the relative increase or decrease in secondary cell wall (SCW) contents compared to the wild type (WT).

**Table 2:** Lignin, cellulose, hemicellulose and pectin contents in *PtoMYB031*-RANi lines.

	WT	*PtoMYB031*-RNAi	
		L2	L8
Lignin			
Acid-soluble	1.48 ± 0.23	1.55 ± 0.05	1.73 ± 0.02
Acid-insoluble	20.67 ± 0.22	20.72 ± 0.46	21.58 ± 0.56* + 4.4%
Total lignin	22.14 ± 0.43	22.27 ± 0.40	23.30 ± 0.57* **+ 5.2%**
Polysaccharide			
Cellulose	44.37 ± 1.80	45.48 ± 1.16* **+ 2.5%**	46.38 ± 0.22* **+ 4.5%**
Hemicellulose	10.34 ± 0.94	11.25 ± 0.29* **+ 8.8%**	12.12 ± 0.49* **+ 17.2%**
Pectin	3.04 ± 0.05	3.54 ± 0.21* + 16.4%	3.63 ± 0.16* **+ 19.4%**

Significance is tested via Student’s t-test, where ‘*’ indicates *p* < 0.05.

The bold values represent the relative increase or decrease in secondary cell wall (SCW) contents compared to the wild type (WT).

**Figure 5:**
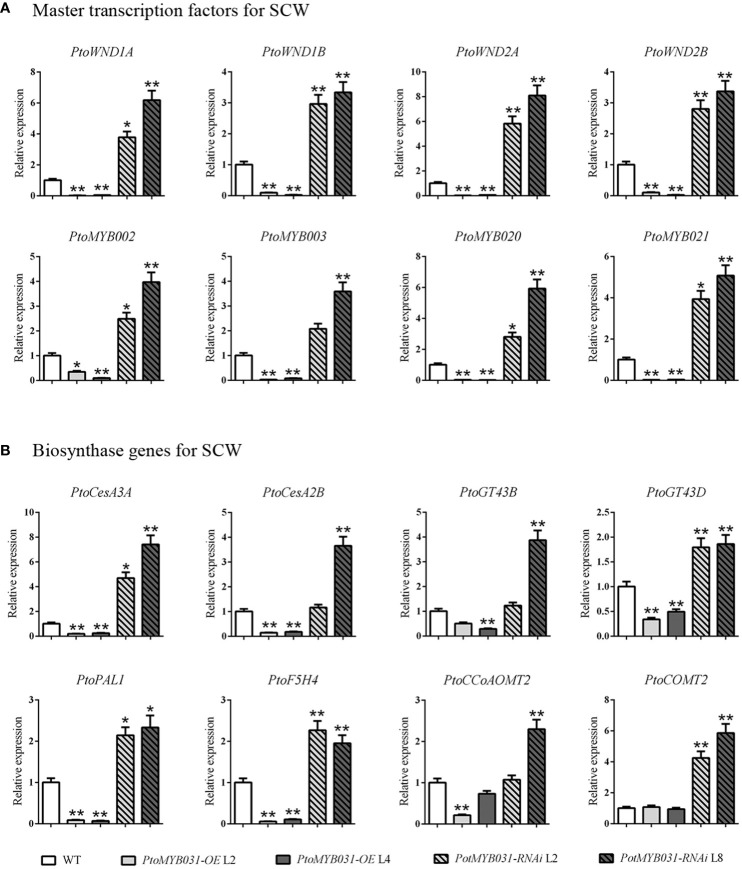
Expression of secondary cell wall-associated genes in WT, *PtoMYB031-*OE, and *PtoMYB031-*RNAi lines. **(A)** Expression profiles of master transcription factors, including SCW-associated NACs (*WND1A*, *WND1B*, *WND2A*, *WND2B*) and SCW-related *MYB* genes (*MYB002*, *MYB003*, *MYB020*, *MYB021*). **(B)** Expression analysis of SCW-associated structural genes, including cellulose biosynthetic genes (*CesA3A* and *CesA2B*), xylan biosynthetic genes (*GT43B* and *GT43D*), and lignin biosynthetic genes (*PAL1*, *F5H4*, *CCoAOMT2*, *COMT2*). Error bars represent standard deviation (± SD) from three biological replicates. Significance tested via Student’s t-test (* *p* < 0.05, ** *p* < 0.01).

To investigate the SCW-related regulatory network, we performed qRT-PCR analysis in *PtoMYB031*-OE lines. The expression levels of the master switch genes (*PtrWND1A/1B/2A/2B*) and secondary switch genes (*PtrMYB002/003/020/021*) in the secondary wall transcriptional regulatory network were decreased (**Figure 5A**). Furthermore, the expression levels of the key enzyme genes involved in lignin biosynthesis (*PAL1*, *4CL5*, and *CCoAOMT*), cellulose synthesis (*CESA2B* and *CESA3A*), and hemicellulose synthesis (*GT43B* and *GT43D*) were also found to be significantly downregulated (**Figure 5B**). In contrast, the *PtoMYB031*-RNAi line showed up-regulation of both SCW-related genes and transcriptional regulatory master switch genes (**Figures 5A, B**). These findings suggested that *PtoMYB031* may function as a negative regulator acting upstream of master switch genes in the SCW regulatory network. Further direct binding experiments are needed to confirm its specific regulatory targets and mechanisms within this pathway.

### Collaboration of *PtoMYB031* and PtoZAT11 in inhibiting secondary cell wall synthesis in poplar

To investigate whether *PtoMYB031* regulates the synthesis of SCW by recruiting other proteins in forming a transcriptional repressor complex, we used PtoMYB031 as a bait in a yeast library screen. The results show that PtoMYB031 may interact with PtoZAT11/12, which contains a canonical EAR repressive motif (**Supplementary Figure S13**). A parallel expression pattern for *PtoMYB031* and *PtoZAT11*, observed within stem sections *via* Aspwood (**Supplementary Figure S14**), suggests a probable complex formation instrumental in xylem morphogenesis.

**Figure 6:**
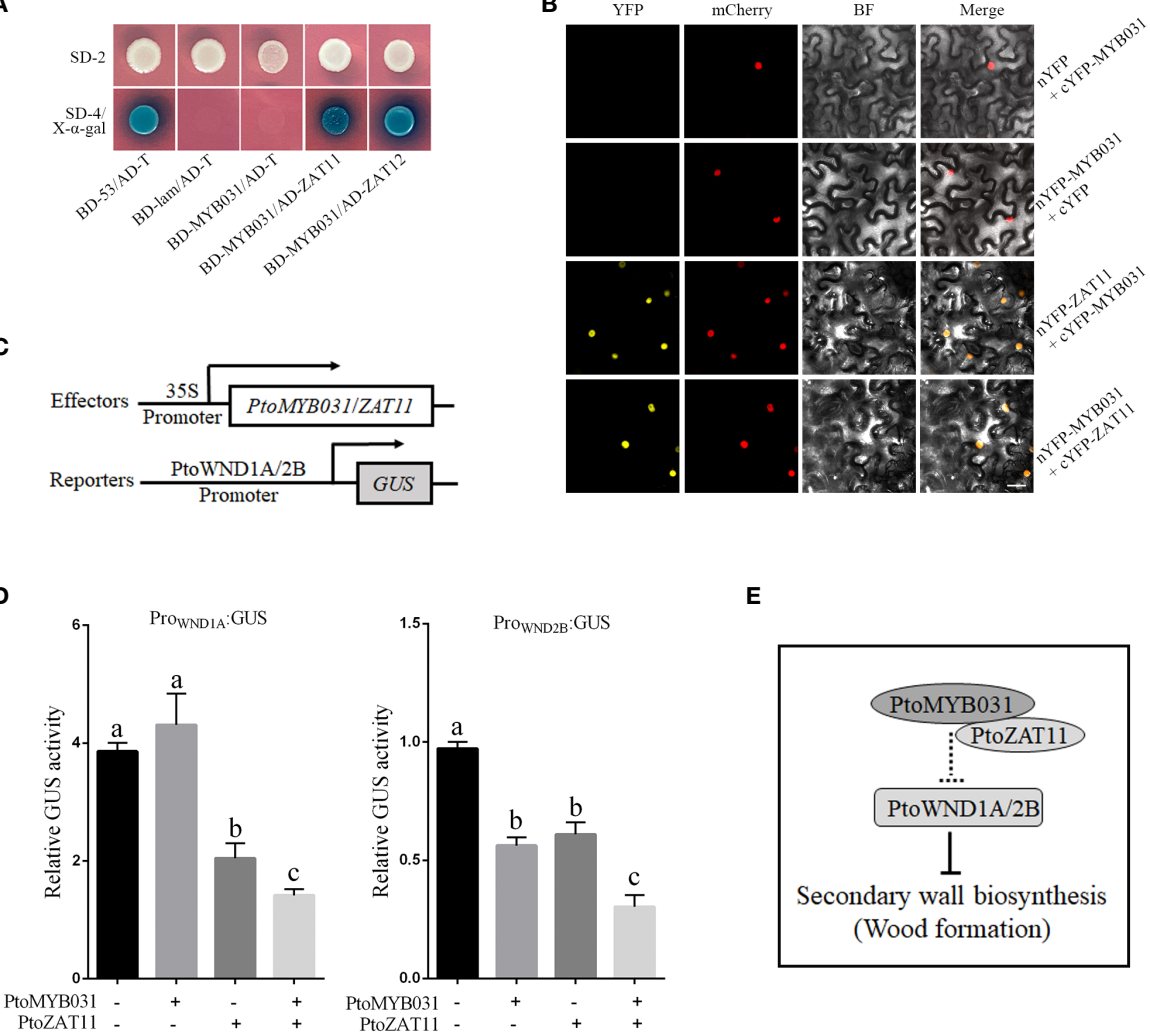
PtoMYB031 interacts with PtoZAT11 to repress the secondary cell wall formation in poplar. **(A)** Yeast two-hybrid (Y2H) assays revealing physical interactions between PtoMYB031 and PtoZAT11/12. PtoMYB031 were fused to the GAL4 binding domain (BD), and PtoZAT11/12 were fused to the GAL4 activation domain (AD). Y2H GOLD yeast strains were co-transformed with pGBKT7-53 + pGADT7-T (positive control), or pGBKT7-Lam + pGADT7-T (negative control). SD/-2 represents the selection medium lacking Trp and leucine (Leu). SD/-4 represents the selection medium lacking Trp, Leu, Ade, and His. **(B)** Protein-protein interaction between PtoMYB031 and PtoZAT11 detected by bimolecular fluorescence complementarity (BiFC) assays. PtoMYB031 and PtoZAT11 was fused with the N-terminus or C-terminus of yellow fluorescent proteins (YFP). The two constructs of PtoMYB031-nYFP and PtoZAT11-cYFP were co-transformed into tobacco leaf epidermal cells. Co-expressions of PtoMYB031-nYFP with cYFP and PtoMYB031-cYFP with nYFP were used as negative controls. The pBI121-mCherry were mixed together at a 1:1 ratio as a nuclear marker. **(C)** Schematic diagram of effector and reporter constructs. The effector contains the coding region of *PtoMYB031* and *PtoZAT11* driven by the *CaMV 35S* promoter. The reporter harbors the *GUS* reporter gene driven by promoters of *WND1A* and *WND2B*. **(D)** Relative GUS activity in transiently transfected tobacco leaves. Significant differences were tested using One-way ANOVA followed by Tukey’s test: different letters represent significant differences. **(E)** Proposed model illustrating *PtoMYB031*’s role in secondary cell wall biosynthesis in poplar.

To substantiate this, we engineered fusion constructs of *PtoMYB031* and *PtoZAT11* with PGBKT7 and PGADT7 vectors, respectively, for yeast two-hybrid assays. Notably, co-transformation of these constructs enabled yeast growth on SD/-4 medium and exhibited blue coloration post x-α-gal interaction, reinforcing the PtoMYB031-PtoZAT11 interaction within the yeast model (**Figure 6A**). Extending our verification to a cellular context, we employed Bimolecular Fluorescence Complementation (BiFC). We designed fusion vectors incorporating *PtoMYB031* with the N-terminal fragment of yellow fluorescent protein (nYFP) and *PtoZAT11* with the C-terminal counterpart (cYFP). Agrobacterium-mediated transient expression in tobacco leaves followed by fluorescence microscopy revealed yellow fluorescence, corroborating the *in vivo* interaction between PtoMYB031 and PtoZAT11 (**Figure 6B**).

Both Y2H and BiFC assays underscore the potential of PtoMYB031 and PtoZAT11 to constitute a transcriptional complex. To discern downstream targets, we employed Effector-Reporter assays. We assembled a *GUS* reporter system under the control of *PtrWND1A*/*1B*/*2A*/*2B* promoters (**Figure 6C**), juxtaposed with effectors *PtoMYB031* and *PtoZAT11* driven by the *35S* promoter. Following transient expression in tobacco leaves, subsequent GUS activity assays revealed that the promoters of *PtrWND1A* and *PtrWND2B* were notably repressed in the presence of the PtoMYB031-PtoZAT11 complex (**Figure 6D**). These insights suggest that PtoMYB031 may interact with PtoZAT11 to form a functional complex that exerts negative regulation on *PtrWND1A* and *PtrWND2B* expression, thereby modulating SCW biosynthesis. This intricate interplay underscores a novel regulatory crosstalk pivotal in dictating SCW formation in poplar. These preliminary findings on the interaction between PtoMYB031 and PtoZAT11, and their impact on PtrWND1A and PtrWND2B, hint at a more complex network of regulatory interactions. For a more comprehensive understanding, further studies are warranted to investigate the broader spectrum of MYB031 interactions, including potential cofactors and other regulatory elements that could influence its activity and the downstream gene expression in the SCW biosynthesis pathway.

## Discussion

Wood formation is a complex biological process. It relies on a transcription network composed of secondary wall NAC and MYB master switches. These switches regulate downstream genes, which encode enzymes vital for the biosynthesis of lignin, cellulose, and hemicellulose (Zhong and Ye, 2015; Chen et al., 2019). The *MYB* superfamily, one of the largest TF families, includes many members identified as critical for wood formation (Xiao et al., 2021). In this study, our study isolated and charaterized a novel R2R3-MYB transcription factor, *PtoMYB031*, from *P. tomentosa*. Overexpression of *PtoMYB031* resulted in a significant reduction in stem diameter and height of the plants (**Figure 3D**). Additionally, there was a decrease in the number of cambial layers, a reduced proportion of xylem, diminished cell wall thickness, and a decline in the content of total lignin, cellulose and hemicellulose (**Figure 4B**; **Supplementary Figure S11**, and **Table 1**). In contrast, the suppression of *PtoMYB031* expression through RNAi led to a increase in the content of secondary wall components (**Table 2**). These findings identify *PtoMYB031* as a pivotal negative regulator in poplar wood formation.

Phylogenetic analysis showed that *PtoMYB031* within the Clade V of *MYB* family (**Supplementary Figure S1**), alongside 12 other poplar and seven *Arabidopsis MYB*s. Investigations into this subgroup’s functional roles have illuminated the genetic and molecular underpinnings of SCW modulation. For instance, previous studies highlighted that dominant repression of *MYB52* significantly diminished SCW thickening, suggesting its repressive role in SCW biosynthesis (Zhong et al., 2008; Cassan-Wang et al., 2013). Notably, poplar homologs *PtrMYB161* and *PtrMYB175* mirror this function, with *PtrMYB161* overexpression in *P. trichocarpa* leading to wood reduction. This phenomenon exemplifies feedback regulation within the *PtrSND1* transcriptional regulatory network (TRN), wherein *PtrMYB161* downregulates all four top-layer regulators and one second-layer regulator (Chen et al., 2019; Wang et al., 2020). For *PtrMYB189*, site-directed deletion and mutagenesis studies have emphasized the critical repression role of its C-terminal 13 amino acids. suggesting the essential role of this region for target repression. A similar repression motif was identified in *PtrMYB158* (Jiao et al., 2019). Intriguingly, although *PtoMYB031* and its closest homolog, *AtMYB69*, both function as transcriptional activators, they exhibit divergent roles in wood formation regulation (Zhong et al., 2008): *AtMYB69* exhibits a minimal impact on secondary cell wall deposition upon overexpression in *Arabidopsis*, while *PtoMYB031* substantially reduces lignin, cellulose, and hemicellulose contents in poplar. Furthermore, *PtoMYB031* overexpression markedly inhibits vascular development in stems, leading to a decrease in SCW thickness, such profound alterations are not observed with *AtMYB69*. This divergence underscores the distinct regulatory roles these transcriptional activators play in wood formation across different plant species.

Most *MYB* repressors, including *AmMYB308*, *AtMYB3*, *AtMYB4*, *AtMYB7*, and *AtMYB32*, localize within the Clade V, collectively exerting negative control over genes encoding enzymes in monolignol and flavonoid pathways. This regulation is attributed to their C-terminal EAR motif, crucial for transcriptional repression (Kranz et al., 1998; Tamagnone et al., 1998; Jin et al., 2000; Preston et al., 2004; Dubos et al., 2010; Ma and Constabel, 2019). Consistently, poplar Clade V members (*PtoMYB156*, *PtrMYB189* and *PdMYB221*), regulated by secondary master switches like *PtrMYB2/3/20/21*, also hinder lignin biosynthesis (Zhong et al., 2013; Tang et al., 2015; Yang et al., 2017; Jiao et al., 2019). This repressive action extends across species, evidenced in *E. gunnii*, *Betula platyphylla*, and *Zea mays*, wherein transcription factors such as *EgMYB1*, *BpMYB4*, *ZmMYB42* and *ZmMYB31* serve as negative regulators (Fornalé et al., 2010; Legay et al., 2010; Yu et al., 2020; Qiang et al., 2022). PtoMYB031, lacking both the C-terminal 13 amino acids found in PtrMYB189 and the EAR motif characteristic of the Clade V, revealed its potential interaction with PtoZAT11/12, possessing a canonical EAR repression motif, through yeast library screening (**Figure 6A**). Further, both BiFC and Y2H assays substantiated the formation of a PtoMYB031-PtoZAT11 transcriptional inhibition complex (**Figures 6A, B**). qPCR analysis elucidated a downregulation of SCW-associated genes, including master switches, in *PtoMYB031-OE* plants and their upregulation in *PtoMYB031-RNAi* lines (**Figure 5**), suggested that *PtoMYB031* upstream in this regulatory hierarchy. Correspondingly, effector-reporter assays demonstrated significant repression of *PtrWND1A* and *PtrWND2B* promoters upon PtoMYB031-PtoZAT11 inreraction (**Figure 6E**).

In summary, our findings revealed the repressive role of *PtoMYB031* in *P. tomentosa* wood formation. Overexpression of *PtoMYB031* negatively influences SCW synthesis, evidenced by the downregulation of associated genes, whereas its RNAi promotes an increase in SCW components, including lignin, cellulose and hemicelluloses. Importantly, PtoMYB031 appears to orchestrate SCW biosynthesis inhibition primarily through the recruitment of the repressor PtoZAT11, forming a transcriptional repression complex. This discovery positions *PtoMYB031* within a complex regulatory network for poplar wood formation, offering valuable insights into the subtle regulatory mechanisms underlying SCW biosynthesis in woody plant species.

## Data availability statement

The datasets presented in this study can be found in online repositories. The names of the repository/repositories and accession number(s) can be found in the article/**Supplementary Material**.

## Author contributions

FT: Data curation, Investigation, Writing – original draft. BJ: Investigation, Writing – original draft, Data curation. MZ: Investigation, Writing – original draft. MH: Investigation, Writing – original draft. RS: Investigation, Writing – original draft. KL: Supervision, Writing – review & editing. TL: Investigation, Writing – original draft, Writing – review & editing.

## Supplementary material

Supplementary Figure 1 Phylogenetic relationship of all the validated poplar *R2R3-MYB* TFs, and their expression patterns. RNA-seq data were sourced from the *Populus* Gene Atlas Study in Phytozome v13.0 (Sreedasyam et al., 2023), with stem-specific expression genes marked by red asterisks.

Supplementary Figure 2 Heatmap visualization of selected *MYB*s expression in developing secondary vascular tissues from the four replicate trees (T1, T2, T3, T4) based on the AspWood database (http://aspwood.popgenie.org/aspwood-v3.0/).

Supplementary Figure 3 Expression pattern of the *PtrMYB026*/*031* gene pair, as presented on the PlantGenIE platform (https://plantgenie.org/exPlot).

Supplementary Figure 4 Protein sequence alignment for Clade V *MYB* genes from *Populus* and *Arabidopsis*. The R2 and R3 regions are highlighted in red, with a focus on amino acid variability and conservation in these regions. Secondary structure predictions (α-helices and β-strands) are depicted as cylinders and arrows.

Supplementary Figure 5 Transcriptional activation and subcellular localization of PtoMYB031. **(A)** Transactivation capability analysis of PtoMYB031. The GAL4BD-PtoMYB031 fusion protein facilitated growth on SD/-1 and SD/-3 (X-α-gal) media, compared to positive (GAL4BD-MYB092) and negative (GAL4BD) controls. SD/-1 and SD/-3 denote medium lacking tryptophan (Trp), and adenine (Ade), histidine (His), and Trp, respectively. **(B)** Localization of PtoMYB031-GFP fusion protein in tobacco (*Nicotiana tabacum*) leaf epidermal cells. The nucleus was indicated by DAPI staining. The *35S::GFP* vector was used as control. Scale bars: 50 µm.

Supplementary Figure 6 Alterations in coding amino acids after gene editing. The occurrence of premature termination proteins in the *ptomyb031* L1 and *ptomyb031* L12 lines as a result of gene editing, in contrast to the wild type (WT).

Supplementary Figure 7 Overexpression of *PtoMYB031* causes upward curl leaf and wrinkle of lamina. The morphology of leaves in *PtoMYB031* OE line 2 and WT. Leaf 2 represent the second leaf from the shoot apical, the same with Leaf 4/6/8. Scale bars: 5 cm.

Supplementary Figure 8 Overexpression of *PtoMYB031* inhibits leaf vascular development. **(A)** Leaf veins staining with phloroglucinol-HCl in *PtoMYB031*-OE L2 and WT. **(B)** Toluidine blue staining at the leaf blade base in *PtoMYB031*-OE L2 and WT. Scale bars: 0.5 cm (First column in **(A)**; 0.1 cm (Second column in A); 200 μm (right columns in **(A)** and all in **(B)**.

Supplementary Figure 9 Comparative microscopic analyses of stem in *PtoMYB031-*OE lines and WT. **(A)** Cross sections from the eighth internode of 3-month-old plants between WT and PtoMYB031-OE line L2. Scale bar: 500 μm. **(B)** Quantitative assessment of pulp and phloem composition, with statistical significance determined using Student’s t-test (* *p* < 0.05, ** *p* < 0.01).

Supplementary Figure 10 Overexpression of *PtoMYB031* inhibits cell wall thickness in stem. Cross-section analysis of stems at the eighth internode in 3-month-old WT (upper) and *PtoMYB031-*OE L2 (lower). Staining methods: Lignin with phloroglucinol-HCl, cellulose with calcofluor. Cell wall thickness was determined using scanning electron microscopy. Xf, xylem fiber; Ve, xylem vessel. Scale bars: 50 μm (left column) and 100 μm (right column).

Supplementary Figure 11 Assessment of cell wall thickness *via* scanning electron microscopy. Cell wall thickness in WT, *ptomyb031* (L1), and *PtoMYB031*-OE lines (L2). Scale bars: 20 μm (upper) and 8 μm (lower). Error bars represent standard deviation (± SD) from three biological replicates (50 counts per each). Significance evaluated using Student’s *t*-test: * *p* < 0.05, ** *p* < 0.01.

Supplementary Figure 12 Growth phenotype and microscopic analyses of stems in *PtoMYB031-*RNAi lines. **(A)** Statistics of stem diameter and xylem area proportion in WT and *PtoMYB031*-RNAi lines. Significance evaluated using Student’s *t*-test: * *p* < 0.05, ** *p* < 0.01. **(B)** Growth phenotype of 3-month-old WT and *PtoMYB031*-RNAi lines (L2 and L8). Scale bars: 10 cm. **(C)** The expression level of *PtoMYB031* and corresponding plant height in WT and *PtoMYB031-*RNAi lines. Statistical significance determined using Student’s *t*-test: * *p* < 0.05, ** *p* < 0.01. **(D)** Cross-section analysis of stems from 3-month-old WT and *PtoMYB031-*RNAi plants. Scale bars: 500 μm.

Supplementary Figure 13 PtoZAT11/12 contains typical EAR (LxLxL) repression motifs. Amino acid alignment for PtoZAT11/12 from *P. tomentosa* and AtZAT11/12 (AT2G37430, AT5G59820) from *A. thaliana*, sourced from the NCBI database. Zinc finger domains are underlined in brown, while EAR motifs (LxLxL) are highlighted in grey with red dashed box.

Supplementary Figure 14 Co-expression analysis of *PtoZAT11* and *PtoMYB031* in Poplar stems. Expression profiles of *PtoMYB031* and *PtoZAT11* in poplar stems, based on data from the Aspwood database. Brown line represents *PtoMYB031*, and green line represents *PtoZAT11*.

Supplementary Data Sheet 1 All sequence alignments used in.

Supplementary Data Sheet 2 All sequence alignments used in **Supplementary Figure S1**.
