# Supplementary figures and images for: PtoMYB031, the R2R3 MYB transcription factor involved in secondary cell wall biosynthesis in poplar
Source: Front Plant Sci. 2024 Jan 17;14:1341245. doi: 10.3389/fpls.2023.1341245 (PMC10828011; doi:10.3389/fpls.2023.1341245)

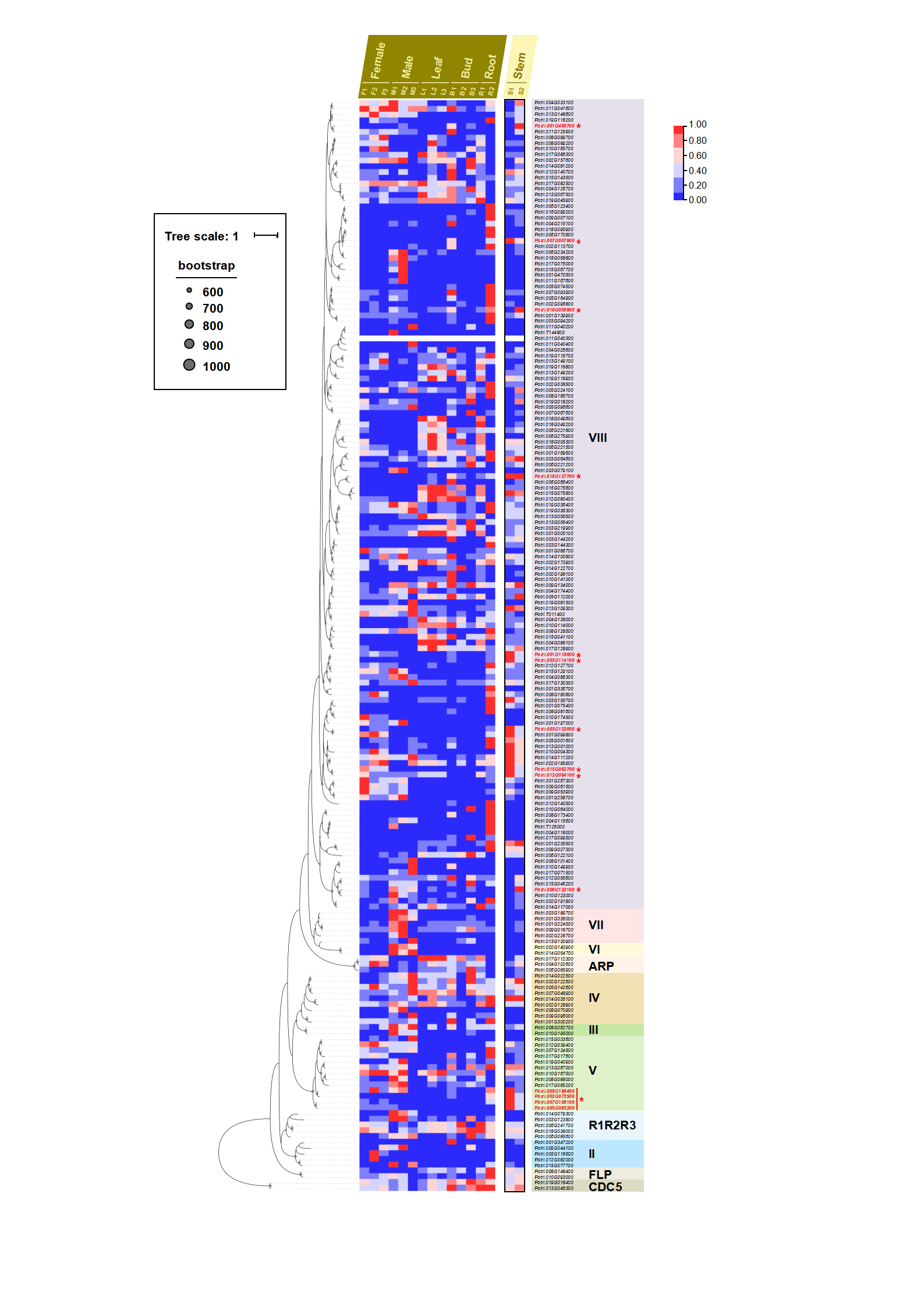

Supplement: Supplementary Figure 1 — Phylogenetic relationship of all the validated poplar R2R3-MYB TFs, and their expression patterns. RNA-seq data were sourced from the Populus Gene Atlas Study in Phytozome v13.0 (Sreedasyam et al., 2023), with stem-specific expression genes marked by red asterisks. [file Image_1.tif]

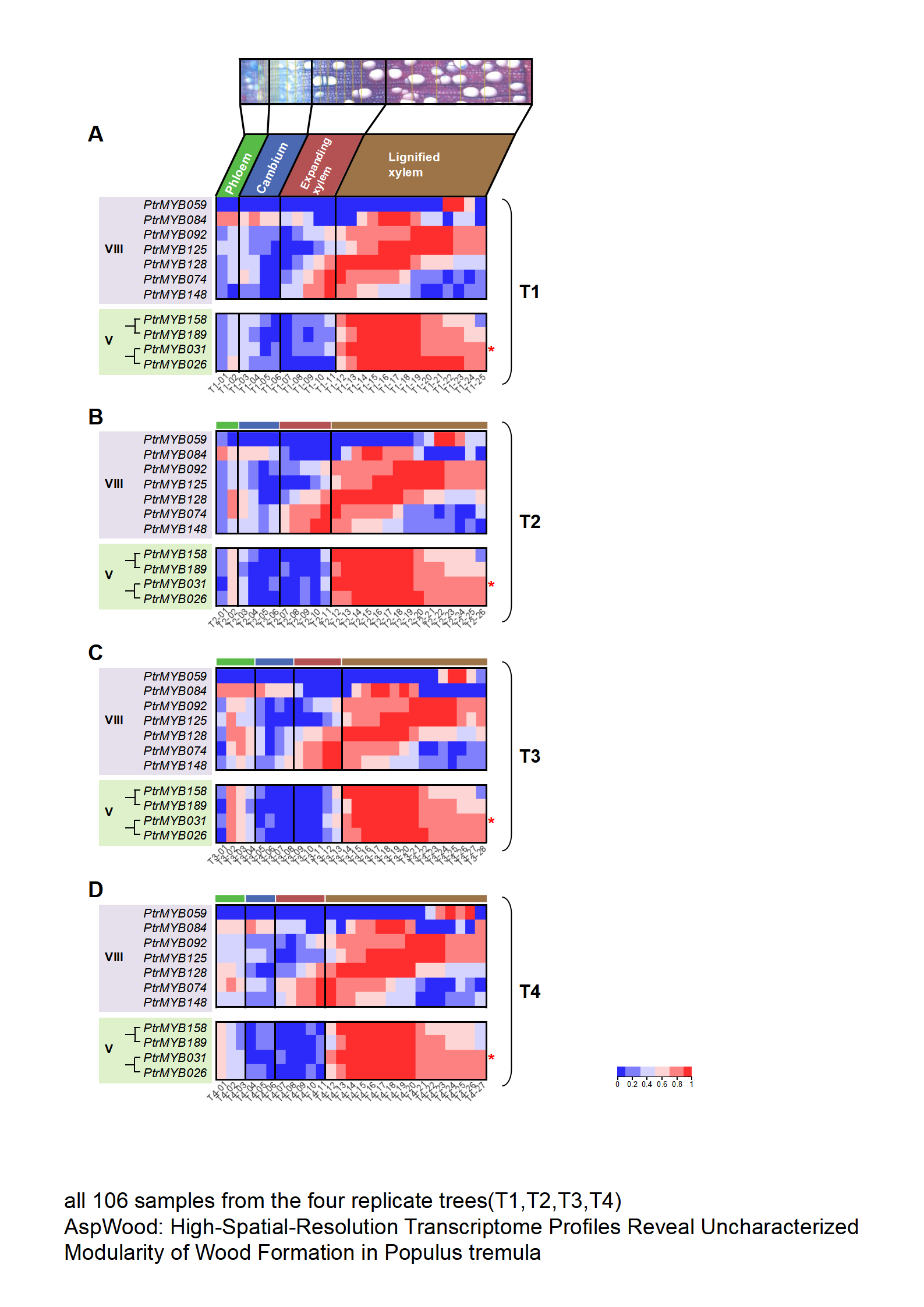

Supplement: Supplementary Figure 2 — Heatmap visualization of selected MYBs expression in developing secondary vascular tissues from the four replicate trees (T1, T2, T3, T4) based on the AspWood database (http://aspwood.popgenie.org/aspwood-v3.0/). [file Image_2.tif]

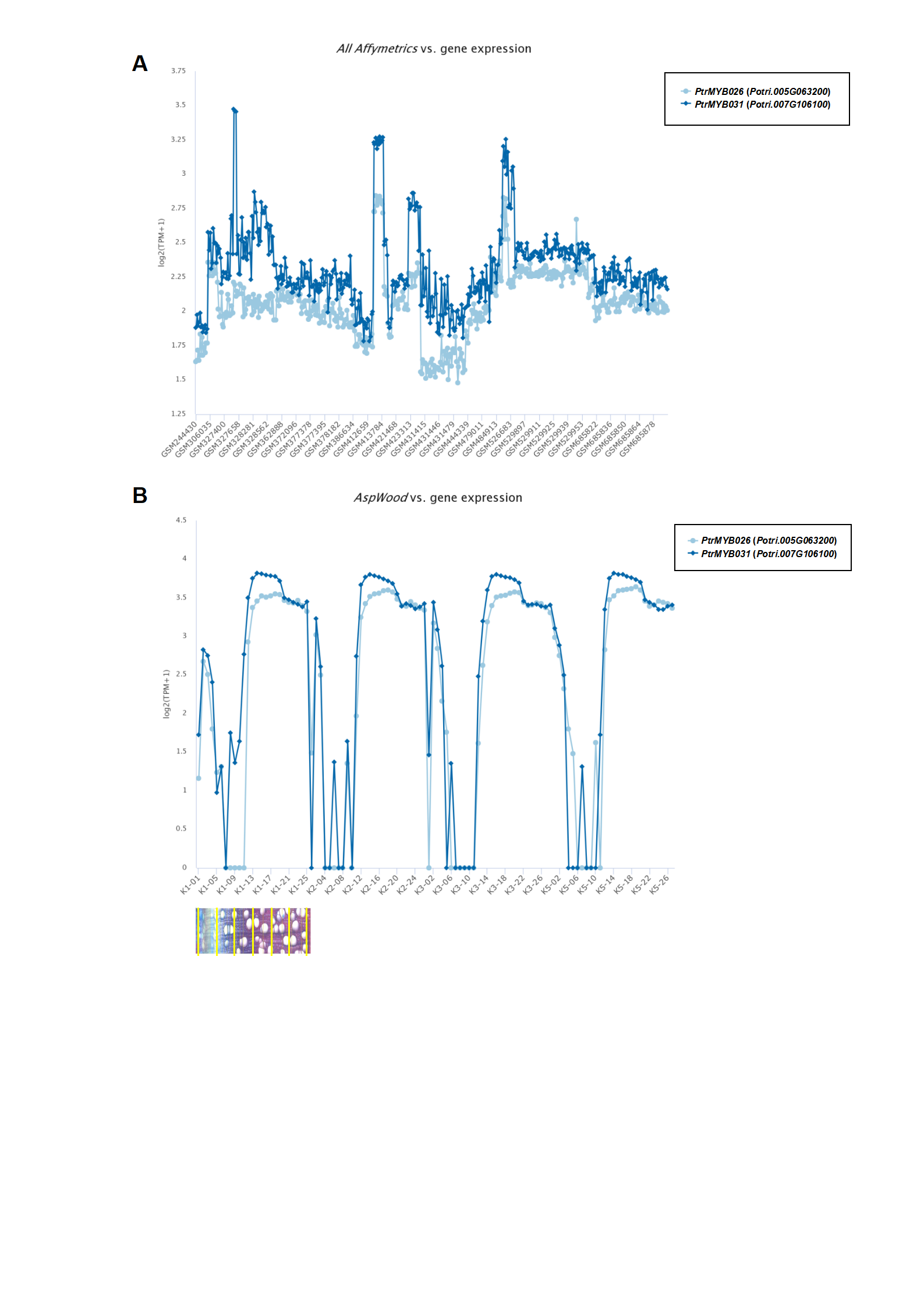

Supplement: Supplementary Figure 3 — Expression pattern of the PtrMYB026/031 gene pair, as presented on the PlantGenIE platform (https://plantgenie.org/exPlot). [file Image_3.tif]

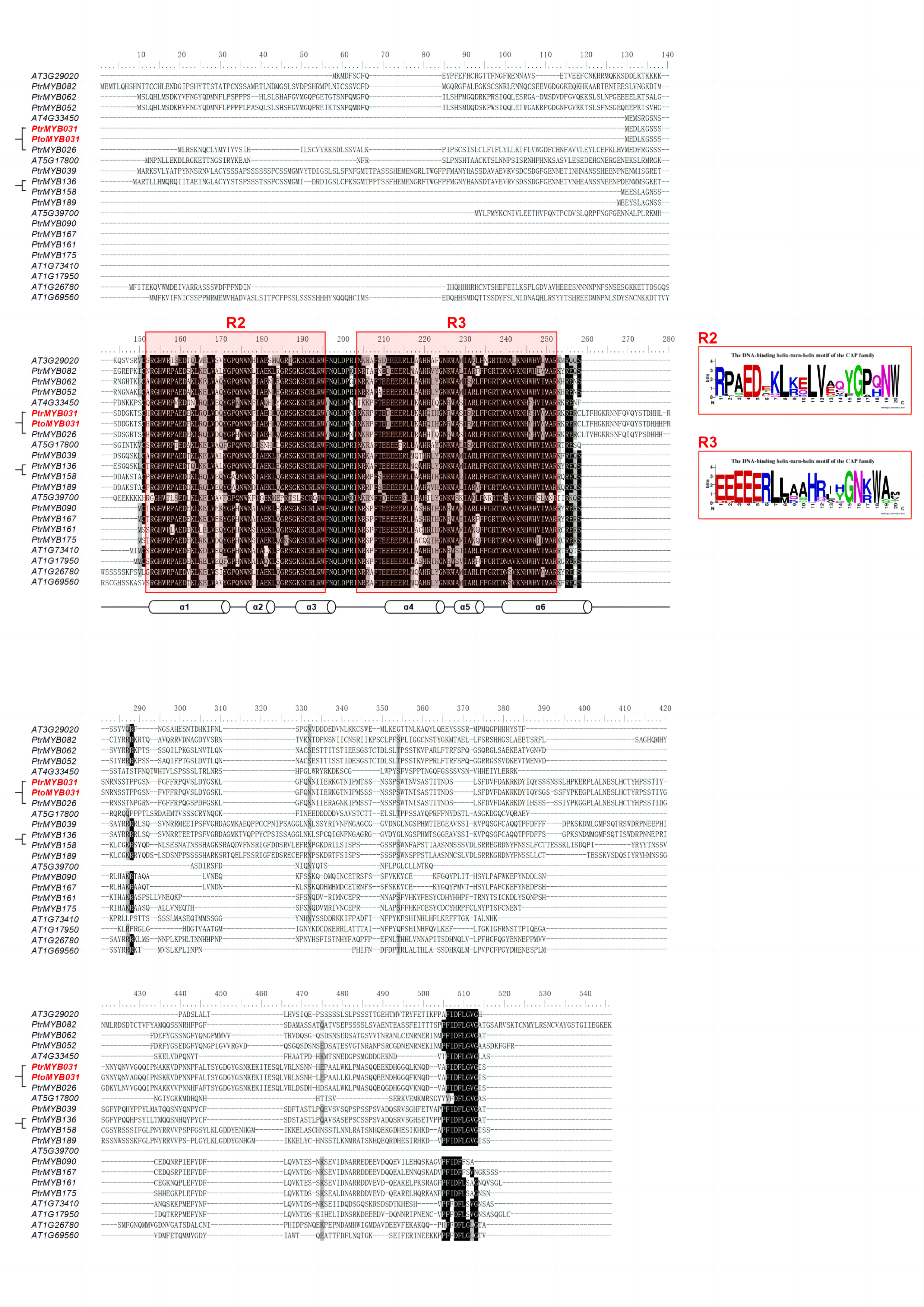

Supplement: Supplementary Figure 4 — Protein sequence alignment for Clade V MYB genes from Populus and Arabidopsis. The R2 and R3 regions are highlighted in red, with a focus on amino acid variability and conservation in these regions. Secondary structure predictions (α-helices and β-strands) are depicted as cylinders and arrows. [file Image_4.tif]

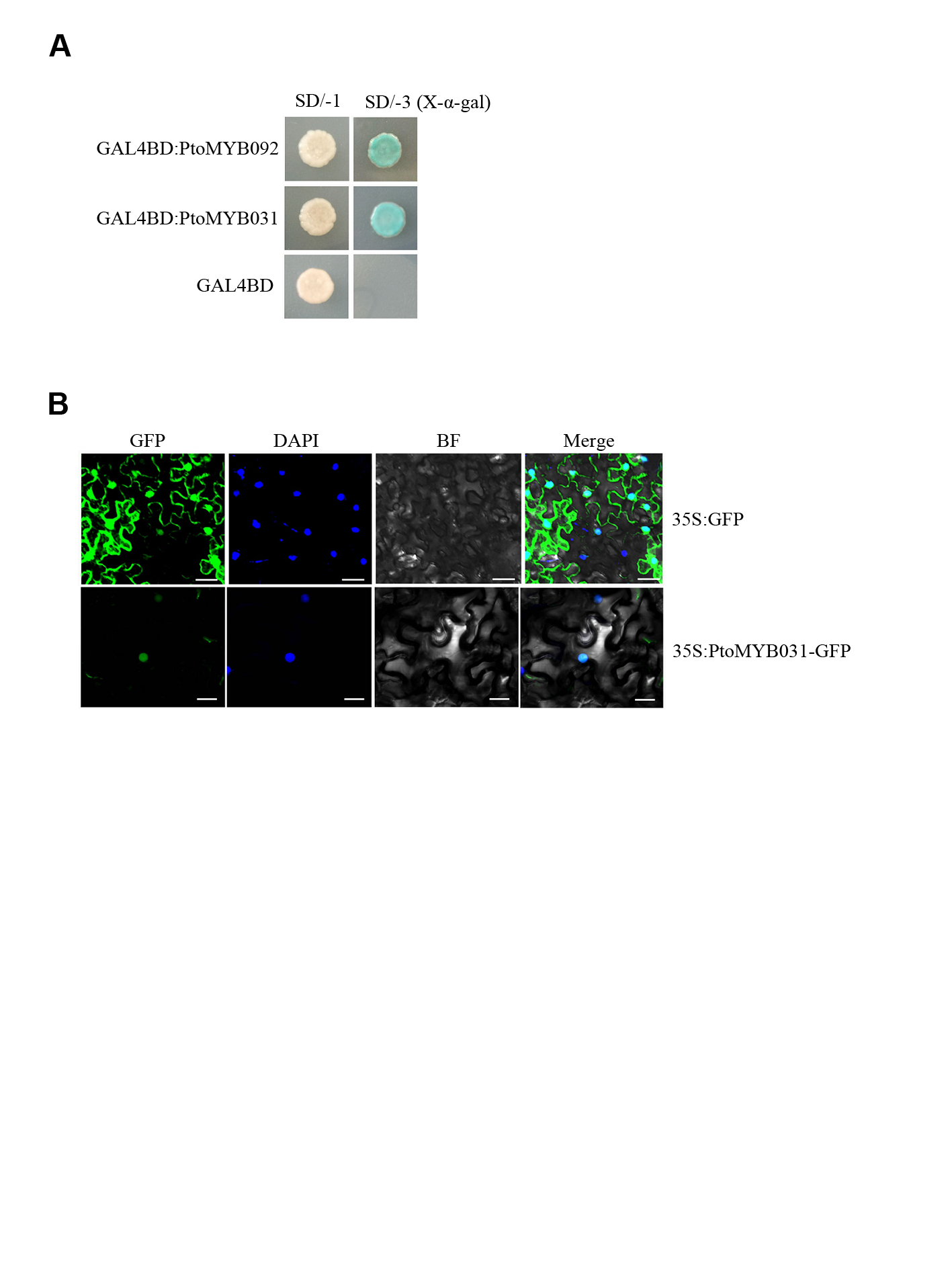

Supplement: Supplementary Figure 5 — Transcriptional activation and subcellular localization of PtoMYB031. (A) Transactivation capability analysis of PtoMYB031. The GAL4BD-PtoMYB031 fusion protein facilitated growth on SD/-1 and SD/-3 (X-α-gal) media, compared to positive (GAL4BD-MYB092) and negative (GAL4BD) controls. SD/-1 and SD/-3 denote medium lacking tryptophan (Trp), and adenine (Ade), histidine (His), and Trp, respectively. (B) Localization of PtoMYB031-GFP fusion protein in tobacco (Nicotiana tabacum) leaf epidermal cells. The nucleus was indicated by DAPI staining. The 35S::GFP vector was used as control. Scale bars: 50 µm. [file Image_5.tif]

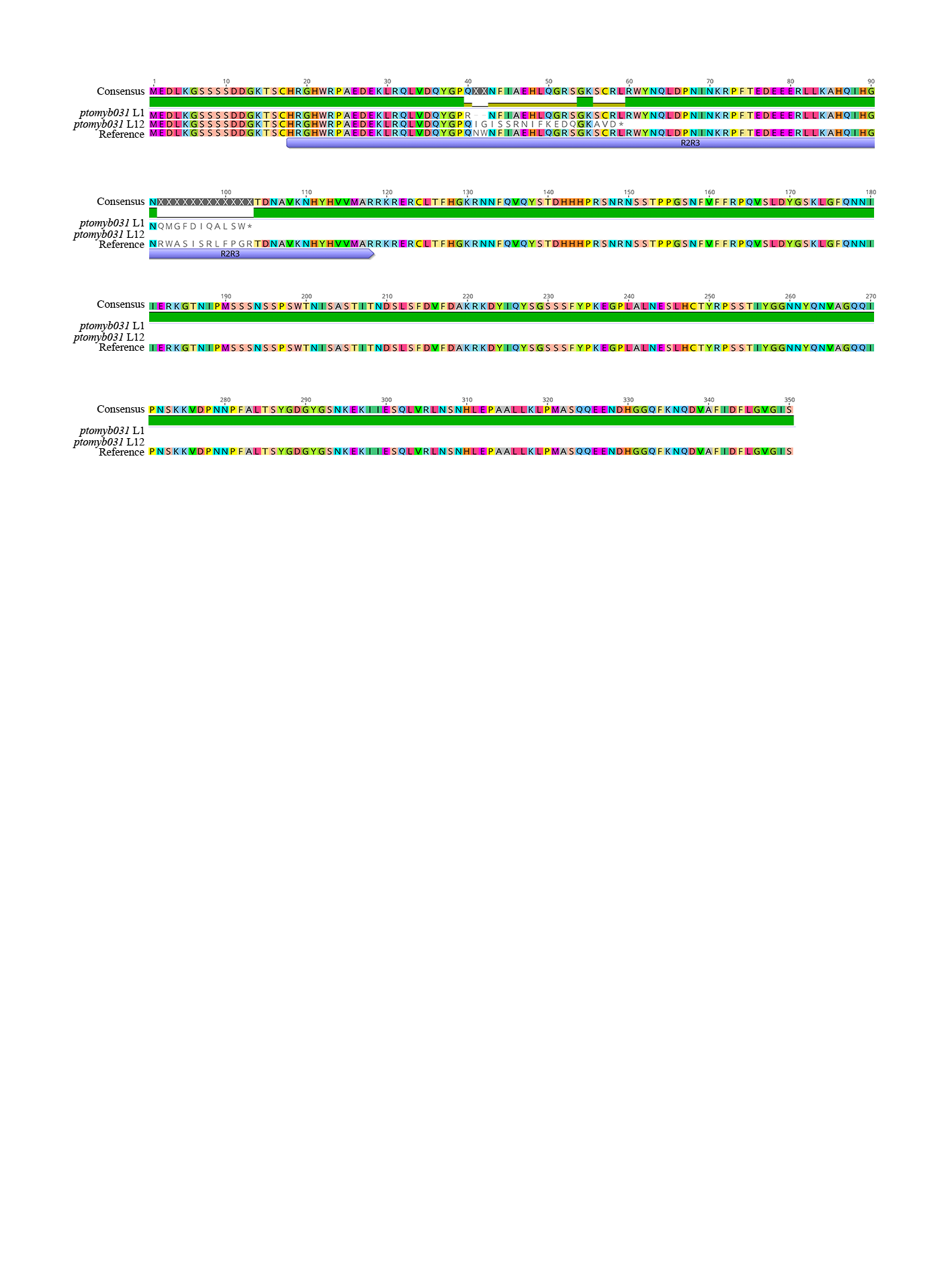

Supplement: Supplementary Figure 6 — Alterations in coding amino acids after gene editing. The occurrence of premature termination proteins in the ptomyb031 L1 and ptomyb031 L12 lines as a result of gene editing, in contrast to the wild type (WT). [file Image_6.tif]

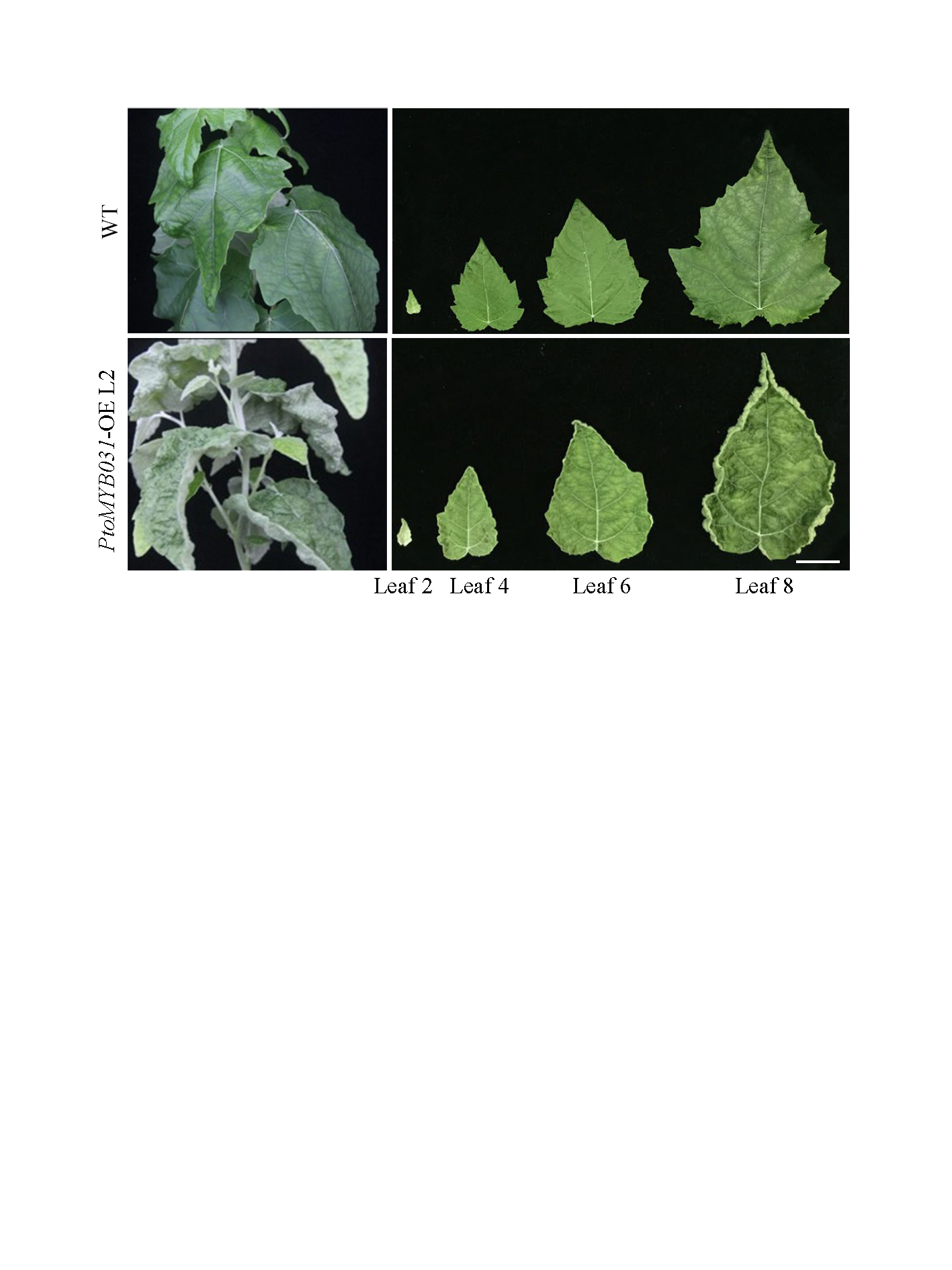

Supplement: Supplementary Figure 7 — Overexpression of PtoMYB031 causes upward curl leaf and wrinkle of lamina. The morphology of leaves in PtoMYB031 OE line 2 and WT. Leaf 2 represent the second leaf from the shoot apical, the same with Leaf 4/6/8. Scale bars: 5 cm. [file Image_7.tif]

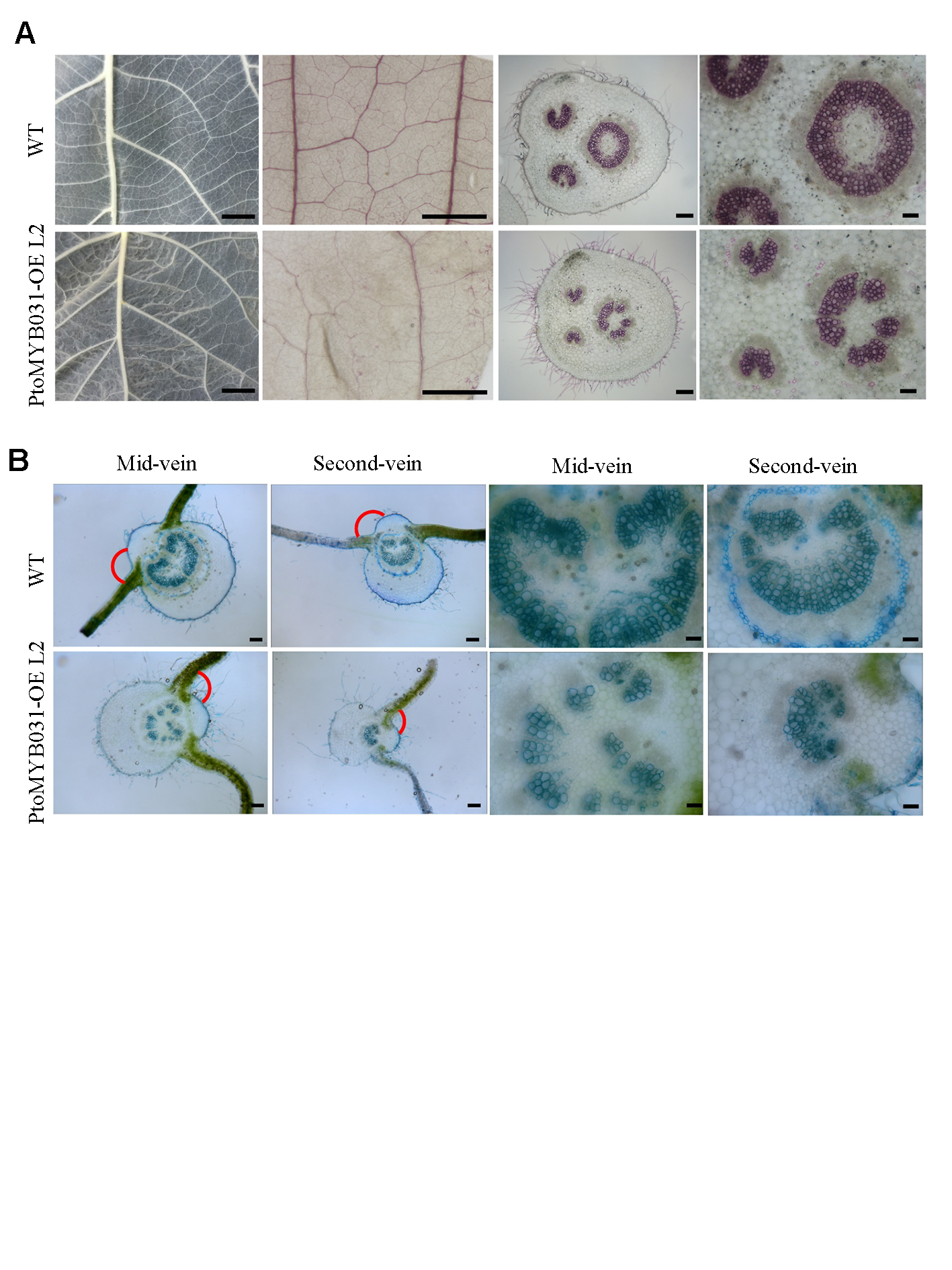

Supplement: Supplementary Figure 8 — Overexpression of PtoMYB031 inhibits leaf vascular development. (A) Leaf veins staining with phloroglucinol-HCl in PtoMYB031-OE L2 and WT. (B) Toluidine blue staining at the leaf blade base in PtoMYB031-OE L2 and WT. Scale bars: 0.5 cm (First column in (A); 0.1 cm (Second column in A); 200 μm (right columns in (A) and all in (B). [file Image_8.tif]

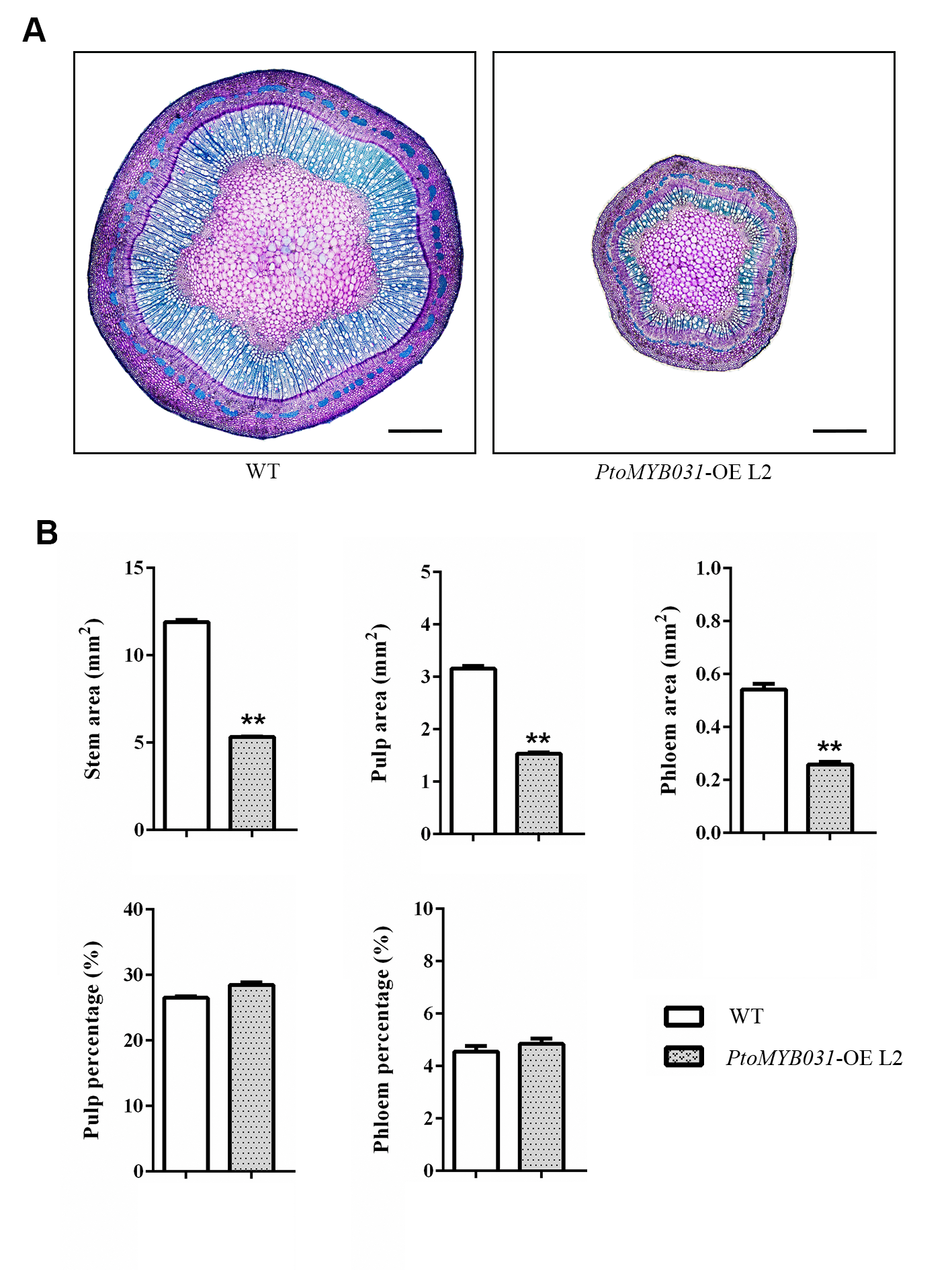

Supplement: Supplementary Figure 9 — Comparative microscopic analyses of stem in PtoMYB031-OE lines and WT. (A) Cross sections from the eighth internode of 3-month-old plants between WT and PtoMYB031-OE line L2. Scale bar: 500 μm. (B) Quantitative assessment of pulp and phloem composition, with statistical significance determined using Student’s t-test (* p < 0.05, ** p < 0.01). [file Image_9.tif]

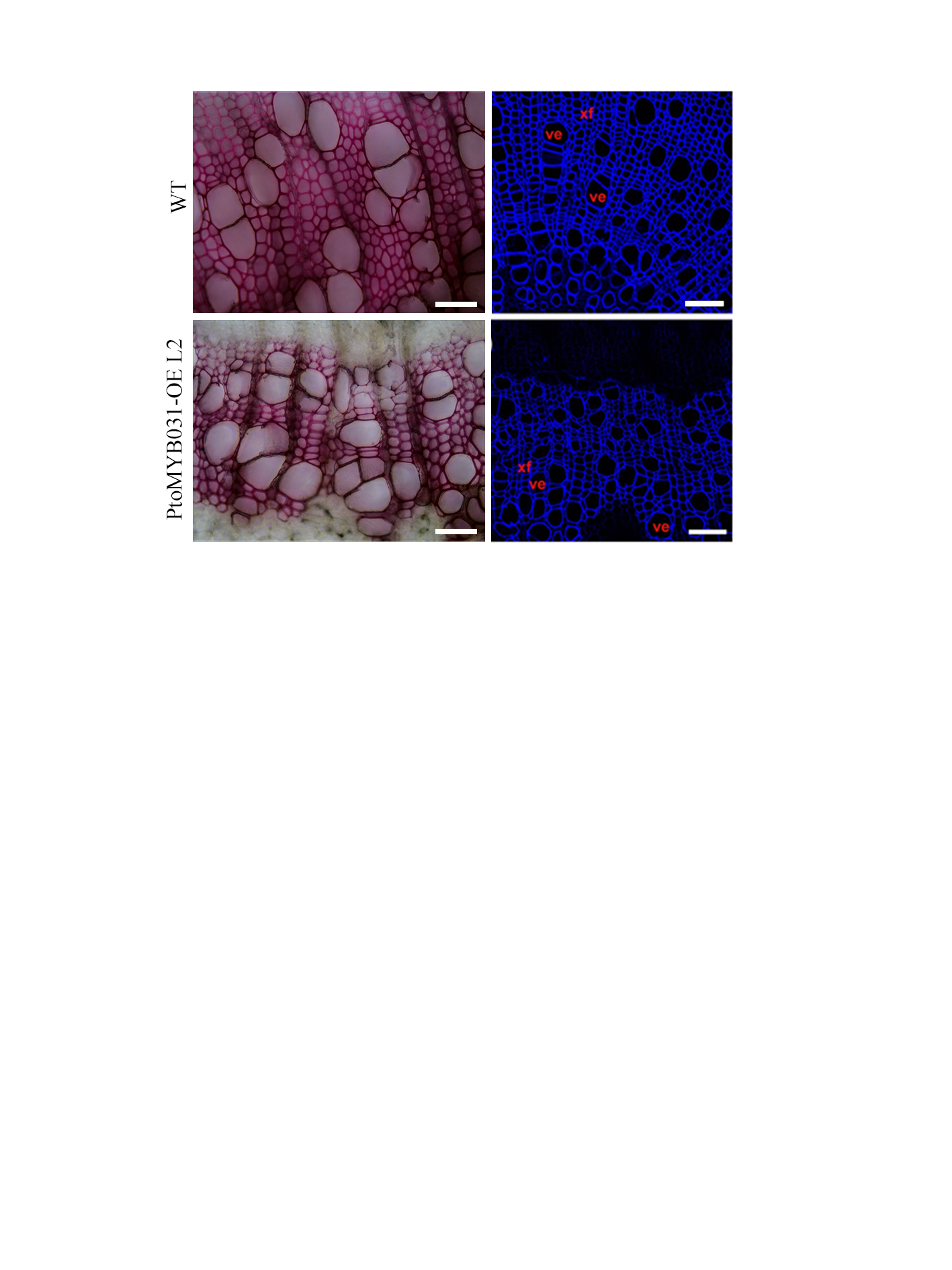

Supplement: Supplementary Figure 10 — Overexpression of PtoMYB031 inhibits cell wall thickness in stem. Cross-section analysis of stems at the eighth internode in 3-month-old WT (upper) and PtoMYB031-OE L2 (lower). Staining methods: Lignin with phloroglucinol-HCl, cellulose with calcofluor. Cell wall thickness was determined using scanning electron microscopy. Xf, xylem fiber; Ve, xylem vessel. Scale bars: 50 μm (left column) and 100 μm (right column). [file Image_10.tif]

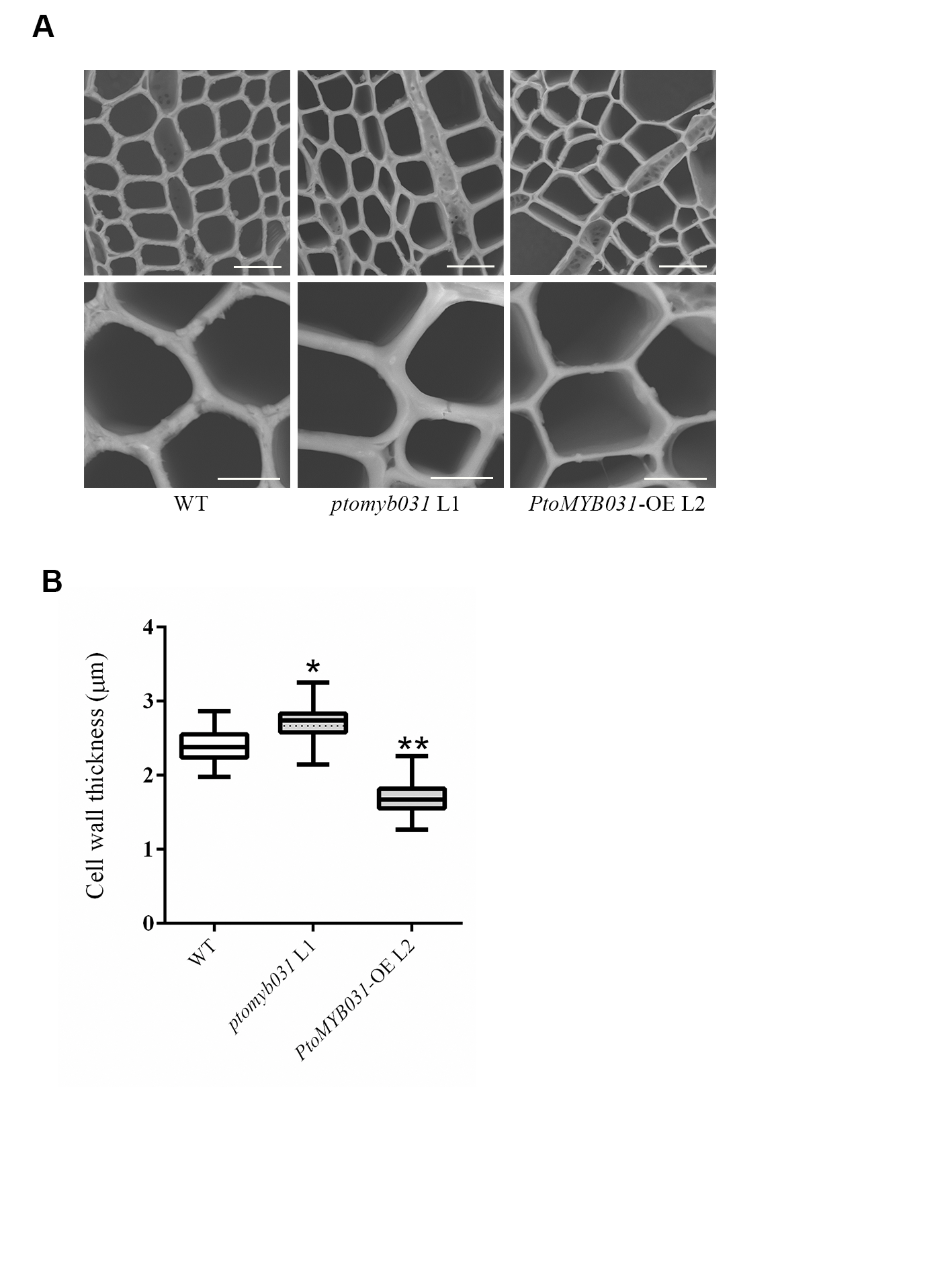

Supplement: Supplementary Figure 11 — Assessment of cell wall thickness via scanning electron microscopy. Cell wall thickness in WT, ptomyb031 (L1), and PtoMYB031-OE lines (L2). Scale bars: 20 μm (upper) and 8 μm (lower). Error bars represent standard deviation (± SD) from three biological replicates (50 counts per each). Significance evaluated using Student’s t-test: * p < 0.05, ** p < 0.01. [file Image_11.tif]

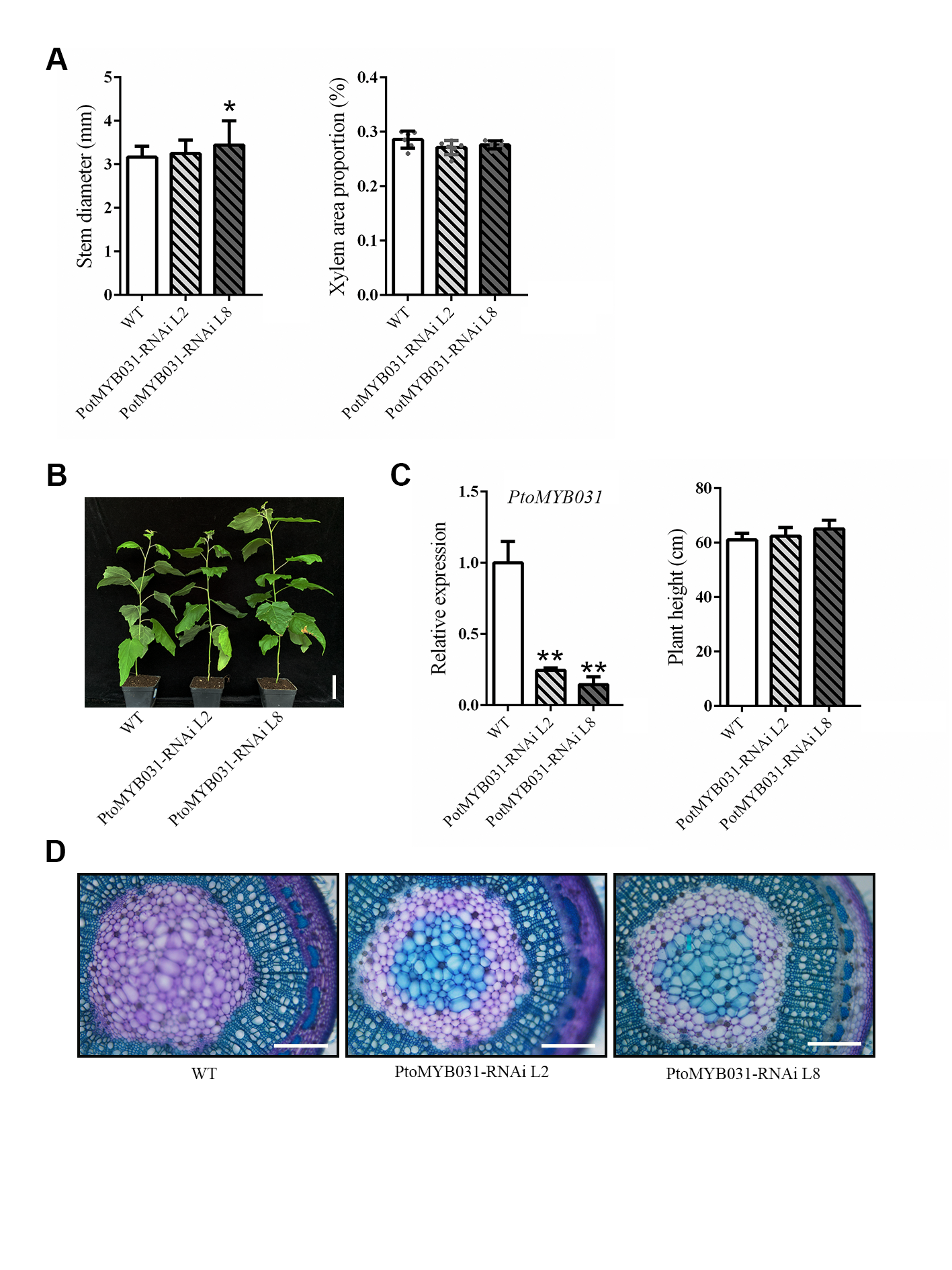

Supplement: Supplementary Figure 12 — Growth phenotype and microscopic analyses of stems in PtoMYB031-RNAi lines. (A) Statistics of stem diameter and xylem area proportion in WT and PtoMYB031-RNAi lines. Significance evaluated using Student’s t-test: * p < 0.05, ** p < 0.01. (B) Growth phenotype of 3-month-old WT and PtoMYB031-RNAi lines (L2 and L8). Scale bars: 10 cm. (C) The expression level of PtoMYB031 and corresponding plant height in WT and PtoMYB031-RNAi lines. Statistical significance determined using Student’s t-test: * p < 0.05, ** p < 0.01. (D) Cross-section analysis of stems from 3-month-old WT and PtoMYB031-RNAi plants. Scale bars: 500 μm. [file Image_12.tif]

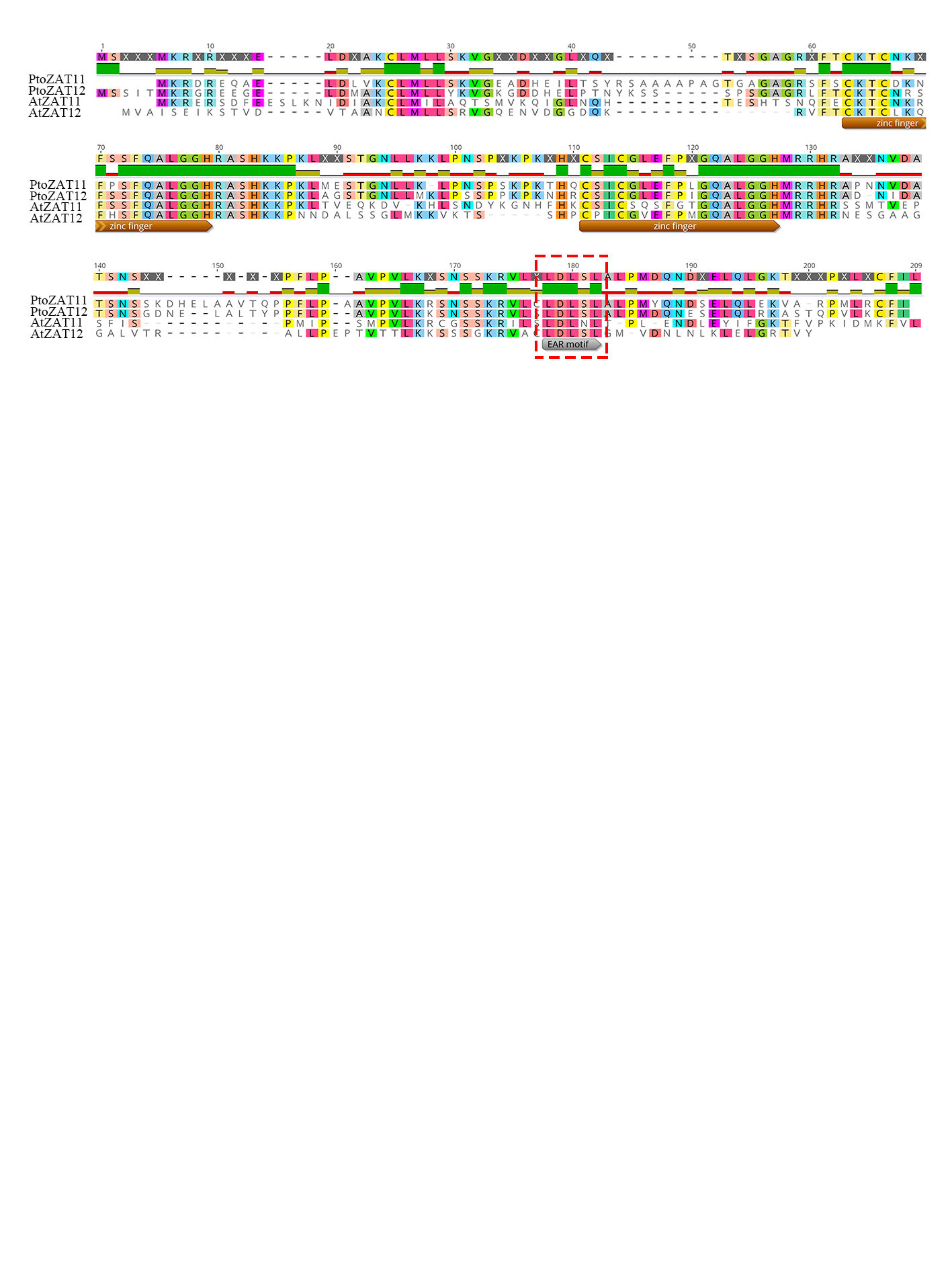

Supplement: Supplementary Figure 13 — PtoZAT11/12 contains typical EAR (LxLxL) repression motifs. Amino acid alignment for PtoZAT11/12 from P. tomentosa and AtZAT11/12 (AT2G37430, AT5G59820) from A. thaliana, sourced from the NCBI database. Zinc finger domains are underlined in brown, while EAR motifs (LxLxL) are highlighted in grey with red dashed box. [file Image_13.tif]

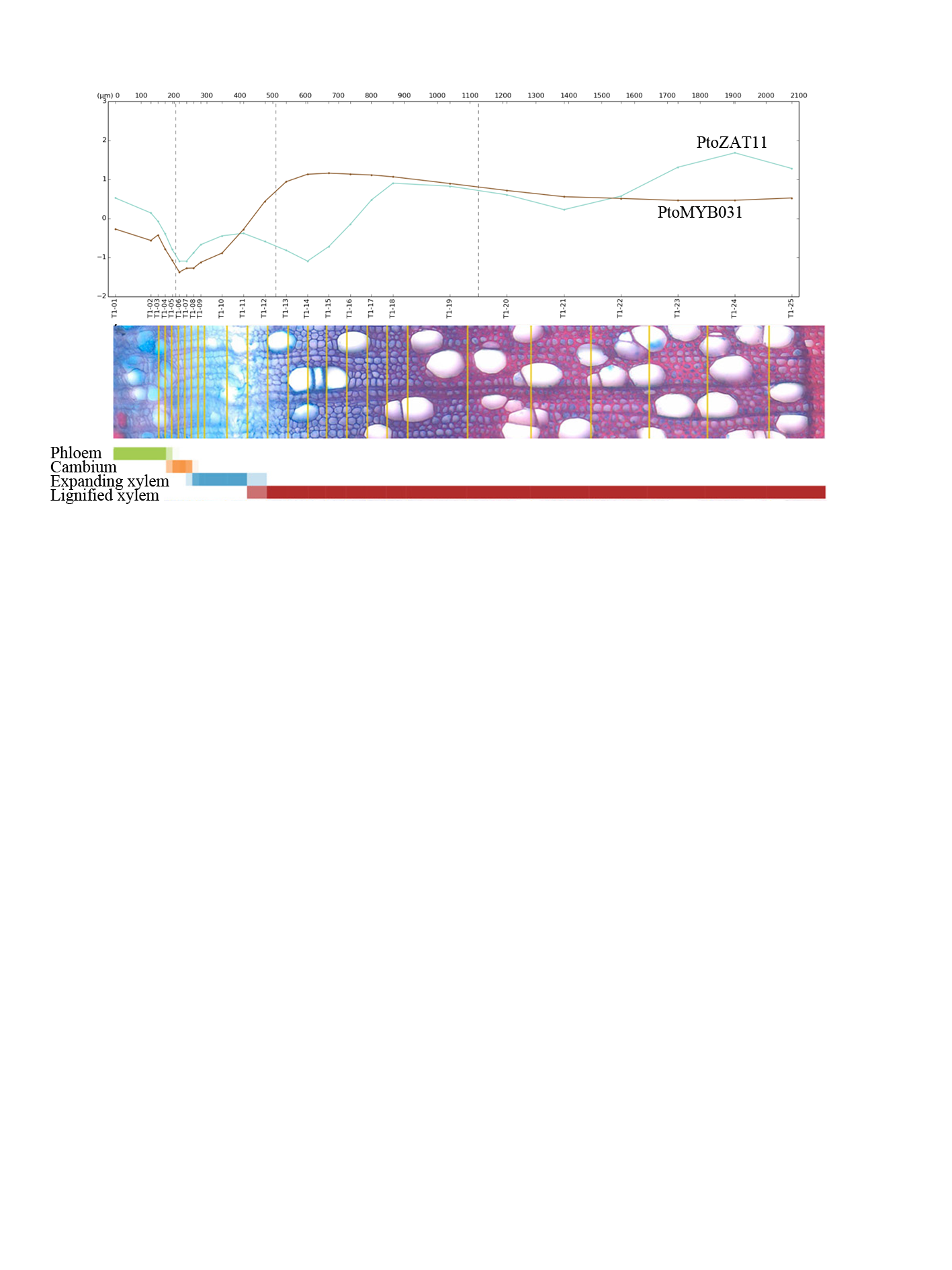

Supplement: Supplementary Figure 14 — Co-expression analysis of PtoZAT11 and PtoMYB031 in Poplar stems. Expression profiles of PtoMYB031 and PtoZAT11 in poplar stems, based on data from the Aspwood database. Brown line represents PtoMYB031, and green line represents PtoZAT11. [file Image_14.tif]
